# “Two birds with one stone” strategy for the lung cancer therapy with bioinspired AIE aggregates

**DOI:** 10.1186/s12951-023-01799-1

**Published:** 2023-02-09

**Authors:** Yinshan Lin, Mengmeng Yi, Xiaoling Guan, Enen Chen, Langyu Yang, Songpei Li, Ying Li, Lingmin Zhang

**Affiliations:** 1 Guangzhou Municipal and Guangdong Provincial Key Laboratory of Molecular Target & Clinical Pharmacology, The NMPA and State Key Laboratory of Respiratory Disease, School of Pharmaceutical Sciences and the Fifth Affiliated Hospital, Guangzhou Medical University, Guangzhou, 511436 China; 2 Key Laboratory of Tropical and Subtropical Fishery Resources Application and Cultivation, Ministry of Agriculture and Rural Affairs; Guangdong Provincial Key Laboratory of Aquatic Animal Immunology and Sustainable Aquaculture Pearl River Fisheries Research Institute, Chinese Academy of Fishery Sciences, Guangzhou, 510380 China

**Keywords:** Bioinspired AIE aggregates, Engineered exosomes, Tumor-associated macrophages, Immunotherapy, Dual targeting

## Abstract

Aggregation-induced emission luminogens (AIEgens) have emerged as novel phototherapeutic agents with high photostability and excellent performance to induce photodynamic and/or photothermal effects. In this study, a zwitterion-type NIR AIEgens C_41_H_37_N_2_O_3_S_2_ (named BITT) with biomimetic modification was utilized for lung cancer therapy. The tumor-associated macrophage (TAM)-specific peptide (CRV) was engineered into the lung cancer cell-derived exosomes. The CRV-engineered exosome membranes (CRV-EM) were obtained to camouflage the BITT nanoparticles (CEB), which targeted both lung cancer cells and TAMs through homotypic targeting and TAM-specific peptide, respectively. The camouflage with CRV-EM ameliorated the surface function of BITT nanoparticles, which facilitated the cellular uptake in both cell lines and induced significant cell death in the presence of laser irradiations in vitro and in vivo. CEB showed improved circulation lifetime and accumulations in the tumor tissues in vivo, which induced efficient photodynamic and photothermal therapy. In addition, CEB induced the tumor microenvironment remodeling as indicated by the increase of CD8 + and CD4 + T cells, as well as a decrease of M2 TAM and Myeloid-derived suppressor cells (MDSCs). Our work developed a novel style of bioinspired AIE aggregates, which could eliminate both lung cancer cells and TAMs, and remodel the tumor environments to achieve an efficient lung cancer therapy. To the best of our knowledge, we are the first to use this style of bioinspired AIE aggregates for photo-mediated immunotherapy in lung cancer therapy.

**Supplementary Information:**

The online version contains supplementary material available at 10.1186/s12951-023-01799-1.

## Introduction

Lung cancer has become one of the fatal cancers worldwide, leading to about 1.6 million deaths each year [1]. The current approaches, such as surgery, chemotherapy, and radiotherapy, showed limited therapeutic effects [2]. One possible reason is that these approaches mainly focus on the single-elimination of cancer cells, which do not reverse the tumor-promoting microenvironment [3]. In the tumor-promoting microenvironment, lung cancer tissues are infiltrated with multiple immune cells, of which tumor-associated macrophages (TAMs) are the most abundant ones [4, 5]. The majority of macrophages in the tumors were M2-like TAMs (also named M2 TAMs), which showed M2 macrophages characteristics, such as tumor-promoting properties, and promoted tumor growth, proliferation, angiogenesis, invasiveness, and metastasis [6, 7]. The elimination of M2 TAMs in the tumors remodeled the tumor environment [8, 9], which was evidenced by the enhanced infiltration of CD4 + and CD8 + T lymphocytes, as well as the reduced myeloid-derived suppressor cells (MDSCs) in tumor microenvironments [10]. The remodeling of the tumor microenvironment has shown great potential in efficient cancer therapy, which reverses the tumor-promoting environment to the tumor-suppressed one and improves immunotherapy by the infiltration of cytotoxic T lymphocytes [11, 12].

Aggregation-induced emission luminogens (AIEgens) possess high brightness, high photostability, enhanced fluorescence intensity, and the ability to generate reactive oxygen species (ROS) in the aggregated state, which are ideal fluorescence materials for image-guided cancer theranostics [13, 14]. Previous work indicated that AIE-based formulations showed great potential in photodynamic therapy (PDT) and/or photothermal therapy (PTT) [15–17]. A zwitterion-type NIR AIEgens C_41_H_37_N_2_O_3_S_2_ (named BITT) showed excellent performance in NIR-II fluorescence imaging-guided synergistic phototherapy against cancer [18, 19]. However, some problems still challenge its applications, such as non-specificity, low stability, or low circulation lifetime, which cause low tumor accumulations and potential side effects on the normal tissues. We consider that ameliorating the surface properties may endow the AIE aggregates with enhanced specificity, long circulation lifetime, and reduced cytotoxicity, which help them cross the biological barrier and achieve efficient lung cancer therapy.

Exosomes are generated through double invagination of the cell plasma membrane and secreted with a size range of ~ 40–160 nm in diameter [20, 21], which have been used as a versatile drug delivery system based on their properties, such as improved stability, long circulation, immunocompatibility, and specificity. However, the direct utilization of exosomes to be vehicles encountered some disadvantages, such as the low loading efficiency and poor plasticity. The camouflage with exosome membrane (EM) has emerged as a promising approach to improve the surface function of nanoparticles. For example, previous work indicated that the camouflage with EM showed better biocompatibility and superior homotypic targeting than cancer membranes [22]. Although tumor-targeting has made some progress based on natural EM, it might not meet the requirements for targeting more expected cell lines. The engineered exosomes based on gene engineering provided the exosomes with specific ligands. For example, the neuron-specific rabies viral glycoprotein (RVG) peptide was fused with lysosome-associated membrane glycoprotein 2b (Lamp2b) and transfected into the cells to obtain RVG-anchored exosomes, which were used to deliver siRNA for the treatment of Alzheimer’s disease [23]. Similarly, the αv integrin-specific iRGD-anchored exosomes were obtained and showed highly efficient targeting of the tumor cells [24]. The evidence suggests that the engineered exosome-derived materials can be used as a platform for drug delivery.

**Fig. 1:**
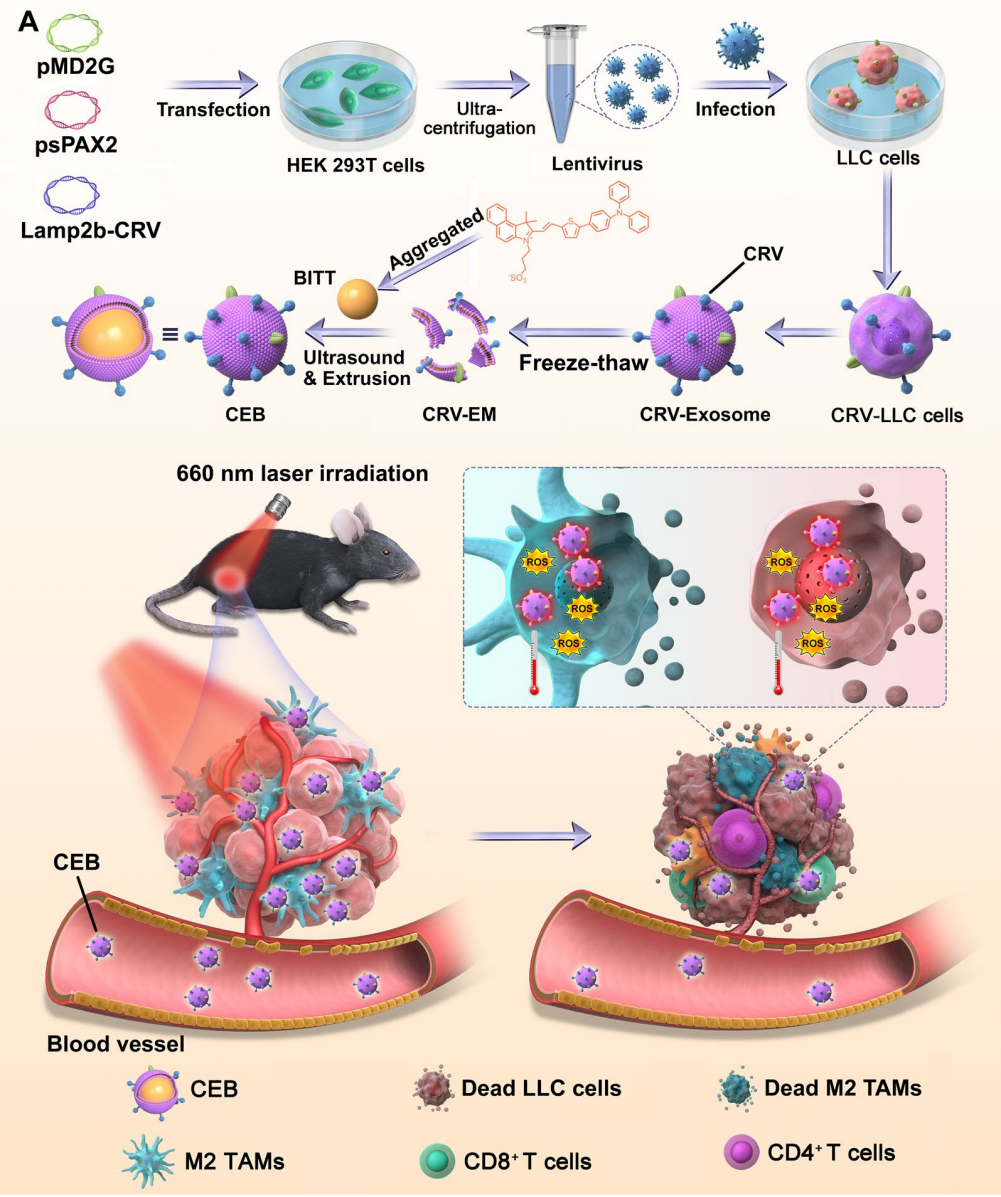
The scheme diagram preparation of CEB and the effect on tumor inhibition. **A** The preparation of CEB. **B** The administration of CEB in vivo

In this study, we developed a dual-targeting strategy to eliminate cancer cells and M2 TAMs to overcome lung cancer. The M2 TAMs targeting peptide, CRV (amino acid sequence, CRVLRSGSC), was overexpressed on the exosomes by fusing CRV sequence into lysosome-associated membrane glycoprotein 2b (Lamp2b) encoded plasmids, which were packaged with lentivirus and transfected in the lung cancer cell line. The exosomes were collected, and CRV-expressed EM (abbreviated as CRV-EM) were used to decorate BITT nanoparticles (abbreviated as CEB) (Fig. 1A) CEB was expected to target both tumor cells and M2 TAMs or M2 macrophages and eliminate them by photodynamic and photothermal therapy in the presence of laser irradiation.(Fig. 1B) Thus, the dual targeting and phototherapeutic system can be considered a novel platform for lung cancer therapy via eliminating cancer cells and remodeling the TME.

## Materials and methods

### Cell culture

Lewis lung cancer cells (LLC), mouse embryonic fibroblast (NIH 3T3), mouse lung epithelial type II cell (MLE12), mouse embryonic fibroblasts (MEF), and human embryonic kidney epithelial cells (HEK 293T) were obtained from the American Type Culture Collection (ATCC, USA). The cells were cultured in DMEM supplemented with 10% fetal bovine serum and 1% penicillin/streptomycin. Cells were kept in an incubation chamber humidified atmosphere at 37 ℃ and 5% CO_2_.

### Construction of CRV-expressed LLC cell line

HEK-293T cells were plated in 150 mm dishes to 30–50% confluence and changed to a transfection medium, Opti-MEM. A volume of 25 μL Lipofectamine 2000 was diluted by 50 μL Opti-MEM and incubated for 5 min. 5 μg pMD2G, 5 μg psPAX2, and 10 μg pcDNA3.1-DNA-Lamp2b-CRV plasmid was mixed gently with 50 μL Opti-MEM medium. The plasmid solution was added to diluted Lipofectamine 2000 and incubated for 20 min. The mixture was added to the culture medium of HEK-293T cells. After the incubation for 6 h, the cells were changed to fresh medium and cultured for 48 h. The medium was collected and changed to fresh media, incubating for another 24 h to do the second harvest. The collected medium was centrifuged at 3 000 rpm for 15 min at room temperature to pellet cell debris. The virus-containing supernatant was filtered through a 0.45 μm and stored at − 80 °C.

LLC cells were plated in a 6-well plate to 30–50% confluence and changed to Opti-MEM containing virus-containing supernatant. After incubation for 6 h, the cells were changed to fresh DMEM supplemented with 10% FBS and cultured for 48 h.

### Isolation of CRV-exosomes and extraction of exosome membranes

For the isolation of the CRV-Exosomes, the CRV-expressed LLC cells were cultured in 150 mm dishes for 48 h. The medium was collected and centrifuged at 3000 g for 5 min at 4 °C to remove cells. The CRV-Exosomes were obtained by centrifuging supernatant at 10,000 g for 15 min at 4 °C followed 100,000 g for 70 min at 4 °C and stored at − 80 °C. A N30 flow nanoanalyzer (NanoFCM, Fujian, China) was used to study the particle size and concentration of the exosomes. The morphology of the exosomes was observed by JEM-2100Plus 200 kV TEM (JEOL, Tokyo, Japan).

To extract the exosomes membranes, the obtained CRV-Exosome precipitation was resuspended in Membrane and Cytosol Protein Extraction Kit reagent A (Beyotime Biotechnology, Shanghai, China) containing 1 mM phenylmethylsulfonyl fluoride. After incubation for 15 min in an ice bath, the mixture was freeze-thawed for 5 cycles. The mixture was centrifuged at a speed of 14,000 g for 30 min at 4 °C. The CRV-EM were quantified with a Pierce BCA Protein Assay Kit and stored at − 80 °C. CRV-expressed EM or EM without CRV were identified by Lamp2b, FLAG, ALIX, and HSP70 through WB analysis.

### Preparation and characterization of CEB

BITT was gifted from Ben Zhong Tang group in The Hong Kong University of Science and Technology. Nuclear Magnetic Resonance (NMR) analysis was performed to identify the molecular structure of BITT. To prepare BITT nanoparticles, BITT was dissolved in the DMSO at a concentration of 1.0 mg/mL, which was added to the aqueous solution at a volume ratio of 1 to 9 in the presence of water bath ultrasonic vibration at a power of 600 W for 5 min. The solution was dialyzed against deionized water with the dialysis bag (MWCO, 3500 Da) for 24 h, which was concentrated by centrifugation at a speed of 14000 g.

To obtain the CRV-EM coated BITT (CEB), BITT nanoparticles was blended with CRV-EM at different ratio (w/w = 1:1, 1:2, 1:5, 1:10, 1:20), followed by 10 times extrusion through 200 nm polycarbonate porous membranes. CEB was analyzed with a Nano ZS90 zetasizer (Malvern, Worcestershire, UK).

The freshly prepared BITT nanoparticles and CEB as described above, were added to carbon-coated copper grids, followed by air drying at room temperature. The analysis was performed with a transmission electron microscope. The UV spectrophotometry, excitation, and emission spectra of BITT and CEB were measured by an UV-1800 spectrophotometer (Shimadzu, Japan) and a RF-6000 fluorospectrophotometer (Shimadzu, Japan).

The protein concentration of CRV-LLC cell membranes and CEB were tested with a Pierce BCA Protein Assay Kit. All samples diluted with SDS-PAGE loading buffer were boiled at 100 ℃ for 5 min. Afterward, each sample with an equivalent protein of 30 μg was loaded onto 10% SDS-PAGE gels and separated via gel electrophoresis. The gel was stained with Coomassie blue for 1 h, followed by washing the gel repeatedly until clear. The data was recorded with an Amersham Imager 600 (GE Healthcare Life Sciences, Little Chalfont, UK).

CEB (BITT equivalent to 15 μg/mL) was irradiated by a LR-MFJ-660/1300mW laser (Changchun laser technology Co., LTD.) for 5 min at different power to evaluate its photothermal conversion efficiencies. Temperature changes were monitored with a FOTRIC 220 s thermal imaging camera (Shanghai InfraRed Systems Co., LTD, China). CEB (BITT ranged 10–20 μg/mL) was irradiated by 660 nm laser for 5 min at the intensity of 2 W/cm^2^. Temperature changes were record as above. BITT and CEB (BITT equivalent to 15 μg/mL) were exposed to a 660 nm laser at the power of 2 W/cm^2^ for 5 min and removed, followed by repeating 4 times. Temperature changes were monitored with the thermographic camera.

The generation of ROS induced by CEB was analysis. Different formulations (BITT equivalent to 15 μg/mL) were incubated with singlet oxygen sensor green (SOSG) probe (5 μM) and exposed to 660 nm laser for 5 min with the power of 2 W/cm^2^. PBS was used as control. The emission spectrums range were scanning at Ex = 504 nm with RF-6000 fluorescence spectrophotometer (Shimadzu, Japan).

### Cellular uptake of CEB in vitro

LLC cells were seeded in confocal dishes or 6-well plates (5 × 10^5^ cells per well). After being cultured overnight, the cells were incubated with CEB at different doses (BITT equivalent to 5, 10, 15, 20 μg/mL) for 6 h, respectively. Cells in the confocal dishes were stained with actin-tracker green and DAPI for visualization by a Zeiss 880 confocal laser scanning microscope (Zeiss, Germany). Cells in 6-well plates were analyzed by an ImageStreamX Imaging flow cytometer (Merck Millipore, USA) to quantify the amounts of BITT-positive cells after digesting by 0.25% tyrisin and resuspending in PBS.

We also evaluated the cellular uptake of CEB over time by CLSM or FACS. Briefly, LLC cells in confocal dishes or 6-well plates were incubated with CEB (BITT equivalent to 15 μg/mL) for different times (1, 3, 6, and 12 h). Cells were stained with actin-tracker green and DAPI for visualization by CLSM. Intracellular fluorescence intensity was quantified by FACS analysis.

After optimizing the dose and time, different formulations, such as BITT, EB, or CEB, were incubated with LLC cells, respectively. CLSM and FACS analysis were applied to monitor the fluorescence.

The cellular uptake of CEB by M2 macrophages was measured. RAW 264.7 cells were seeded either in confocal dishes or in 6-well plate (2 × 10^5^ cells per well) and treated with IL4 (50 ng/mL) for 48 h. BITT (concentration range 5–20 μg/mL) was added to the culture medium, and cells were cultured for 1, 3, 6, and 12 h. Cells were observed upon CLSM after being stained by actin-tracker green and DAPI or calculated positive cells by FACS. The induced M2 macrophages were incubated with BITT, EB, or CEB according to the optimized condition. The fluorescence was captured by CLSM and FACS.

CEB was prepared as described above. CEB (BITT equivalent to 15 μg/mL) was incubated with non-specific cell lines, including NIH 3T3 cells, MLE12 cells, and MEF cells for 6 h at 37 °C. The cells were washed three times with PBS. For CLSM analysis, the cells were fixed and stained with actin-tracker green and DAPI. To quantitatively analyze cellular uptake, the adherent cells were detached from the culture plate with 0.25% Trypsin–EDTA Solution and suspended in 100 μL PBS. The cell suspension was analyzed by FACS.

### Assessment of ROS generation in cells

The ROS generation induced by BITT-based formulations was detected by the reactive oxygen species assay kit. LLC cells and M2 macrophages cultured in confocal dishes were treated with PBS, PBS with Laser (PBS + Laser), CEB, BITT with Laser (BITT + Laser), EB with Laser (EB + Laser), and CEB with Laser (CEB + Laser) for 6 h. DCFH-DA diluted in FBS-free DMEM to 10 μM was incubated with the cells at 37 °C for 30 min in the dark. FBS-free DMEM washed cells to remove DCFH-DA that did not enter cells. Groups with laser were irradiated by a 660 nm laser (2 W/cm^2^, 5 min). CLSM was applied to observe the fluorescence at Ex = 488 nm and Em = 525 nm.

### Cell viability in vitro

The cells viability of BITT based nanoparticles with or without laser irradiations was measured by cell counting kit 8 (CCK-8) assay. LLC cells and polarized M2 macrophages were seeded in 96-well plate (5 × 10^3^ cells/well) and treated with different formulations (BITT equivalent to 0 ~ 20 μg/mL) for 6 h. Cells were performed with or without irradiation by laser (660 nm, 2 W/cm^2^) for 5 min. After incubation for 4 h, the medium was discarded, and 10% CCK-8 solution was added. After incubation for another 2 h, the absorption at 450 nm was measured. The viability was calculated by the formula:

$${\text{Cell viability }}\left( \% \right)\, = \,\left[ {\left( {{\mathrm{A}}_{{{\mathrm{sample}}}} - {\mathrm{A}}_{{\text{blank well}}} } \right)/ \, \left( {{\mathrm{A}}_{{{\mathrm{control}}}} - {\mathrm{A}}_{{\text{blank well}}} } \right)} \right] \, *{1}00\% .$$

### Cell inhibition in vitro

LLC and M2 macrophages were seeded in confocal dishes and cultured overnight. PBS, PBS + Laser, CEB, BITT + Laser, EB + Laser, or CEB + Laser was added to the cells. After incubation for 6 h, the cells were exposed to a 660 nm laser for 5 min at the intensity of 2 W/cm^2^. After the cells were cultured for another 4 h, cells were stained with Calcein-AM and PI for 15 min, the live and dead cells were imaged by CLSM.

### The evaluation of CEB in 3D tumor spheroids

Agarose was dissolved in DMEM to obtain a 2% solution. The solution (60 μL) was used to coat the bottom 96-well plate. LLC cells were seeded in the agarose-coated plate (4 × 10^3^ cells/well) and cultured for 5 days. After forming ~ 500 μm 3D tumor spheroids, the tumor spheroids were transferred to the agarose-coated confocal dished and treated with BITT, EB, or CEB for 24 h. The cellular spheroids in confocal dished were stained with DAPI for 30 min and imaged by the Z-stack function of CLSM.

To test the phototherapeutic effect on 3D tumor spheroids, the spheroids were incubated with different formulations (PBS, PBS + Laser, CEB, BITT + Laser, EB + Laser, CEB + Laser) for 24 h, and further exposed to a 660 nm laser for 5 min at the intensity of 2 W/cm^2^. The spheroids were incubated in the DMEM completed medium for another 4 h. Live/Dead Cell Viability Assay Kit was used to stain the spheroids and imaged with a CLSM.

### Animal experiments

The 4 ~ 6-weeks-old C57BL/6 male mice were purchased from Guangdong Medical Laboratory Animal Center (Foshan, China) and raised in the specific pathogen-free animal room. We performed the animal experiments according to the Institutional Authority for Laboratory Animal Care of Guangzhou Medical University (GY2021-142).

### Evaluation of circulation lifetime in vivo

The C57BL/6 male mice were administrated with different formulations (PBS, BITT, EB, or CEB; n = 3, BITT equivalent to 200 μg per mouse) via intravenous tail vein injections. A volume of 20 μL of whole blood was collected at time points 1, 2, 4, 6, 8, 12, and 24 h. The samples were mixed with 0.1 mM acid citrate dextrose and kept at 4 °C until detected by a fluorescence spectrophotometer.

### Distribution of CEB in vivo

The C57BL/6 male mice were subcutaneously inoculated with 3 × 10^6^ LLC cells per mouse on the right shoulder to construct the tumor-bearing mouse model. When tumor volumes reached approximately 100 mm^3^, the mice were administered with PBS, BITT, EB, or CEB (BITT equivalent to 200 μg per mouse) via intravenous tail vein injections and monitored with an IVIS Lumina XRMS Series III in vivo tracking system (PerkinElmer, USA). The time points were set at 2, 4, 8, 12, 24, and 48 h, respectively. The mice were sacrificed, and the tumors and major organs were extracted and imaged. The fluorescence was analyzed with Living image software.

### Antitumor effect of CEB in vivo

The xenograft tumor mouse model was constructed by subcutaneously inoculating with 3 × 10^6^ LLC cells per mouse on the right flank of C57BL/6 male mice. When the tumor volumes reached approximately 100 mm^3^, the mice were randomly divided into six groups (n = 5 per group): PBS, PBS + Laser, CEB, BITT + Laser, EB + Laser, CEB + Laser (BITT equivalent to 200 μg per mouse). The mice were administered different formulations via intravenous tail vein injections every other day and received a 660 nm laser irradiation (2 W/cm^2^, 10 min) 8 h later. The tumor volumes were measured with a Vernier caliper and calculated as V = (L × W × W)/2. The weight of mice was monitored as well. After the treatment for 13 days, the mice were sacrificed to collect organs and tumors. Tumors were arranged in orderliness and photographed. The major organs and tumors were stored in 4% paraformaldehyde and analyzed with Hematoxylin and eosin staining (HE) and TdT-mediated dUTP Nick-End Labeling (TUNEL).

### The effect of CEB on tumor microenvironment remolding

To determine the in vivo remodeling of tumor microenvironment, the amount of M1 and M2 TAMs, CD8^+^ T cells, CD4^+^ T cells, and MDSCs were determined by FACS and CLSM analysis. Briefly, tumor-bear mice were treated with different formulations as described above. After the treatments, the mice were sacrificed, and the tumors were cut into pieces and digested with collagenase IV at 37 °C for 20 min under vibrating. The mixtures were homogenized with a 40 μM strainer. After the lysis of red blood cells, cells were washed with HBSS and divided into 3 tubes. The APC-Cy7-anti-CD45, PE-anti-F4/80^+^, BV421-anti-CD80, and AF647-anti-CD206 antibodies were added to the collected cells for 30 min on ice. The staining solutions were removed and washed with PBS, followed by fixing the cells with 4% paraformaldehyde. To analyze the T cells, APC-Cy7-anti-CD45, BV510-anti-CD3e, FITC-anti-CD8a, and APC-anti-CD4 antibodies were added to the cells and incubated on ice for 30 min. MDSCs were identified by the addition of APC-Cy7-anti-CD45, PE-anti-Gr1, and Percp-Cy5.5-anti-CD11b antibodies on the ice for 30 min as well. All the samples were analyzed by FACS.

Furthermore, the tumors were cut into 4-μm-thick sections and tested by immunofluorescence. M1 and M2 TAM were labeled with anti-CD80 and anti-CD206 antibodies. Anti-CD8 and anti-CD4 antibodies were applied to label T cells. MDSCs were identified with anti-Ly6G and anti-CD11b antibodies. Interestingly, the suppression of vascularization was detected by anti-CD31 and anti-α-SMA antibodies. Immunofluorescence sections were imaged by CLSM.

### Statistical analysis

The data are expressed as the mean ± standard deviation. Data analyses were conducted using GraphPad Prism 8.0 software. A two-tailed Student’s *t* test for a two-group comparison was used to analyze the data. Statistical differences are shown as *p < 0.05, **p < 0.01, and ***p < 0.001.

## Results and discussion

### The preparation and characterization of CEB

We prepared and characterized CEB . Firstly, to acquire cancer cells exosomes that target to both LLC cells and M2 macrophages, LLC cells were transfected with lentiviral packaged Lamp2b-CRV-encoded plasmid (Additional file 1: Fig. S1). The CRV-expressed cancer cells exosomes (CRV-Exosomes) were isolated and spherical with a size less than 100 nm (Additional file 1: Fig. S2). The CRV-Exosome membranes (CRV-EM) were collected. The CRV-EM was identified by the increased expression of Lamp2b, FLAG tag, ALIX, and HSP70 (Additional file 1: Fig. S3).

**Fig. 2:**
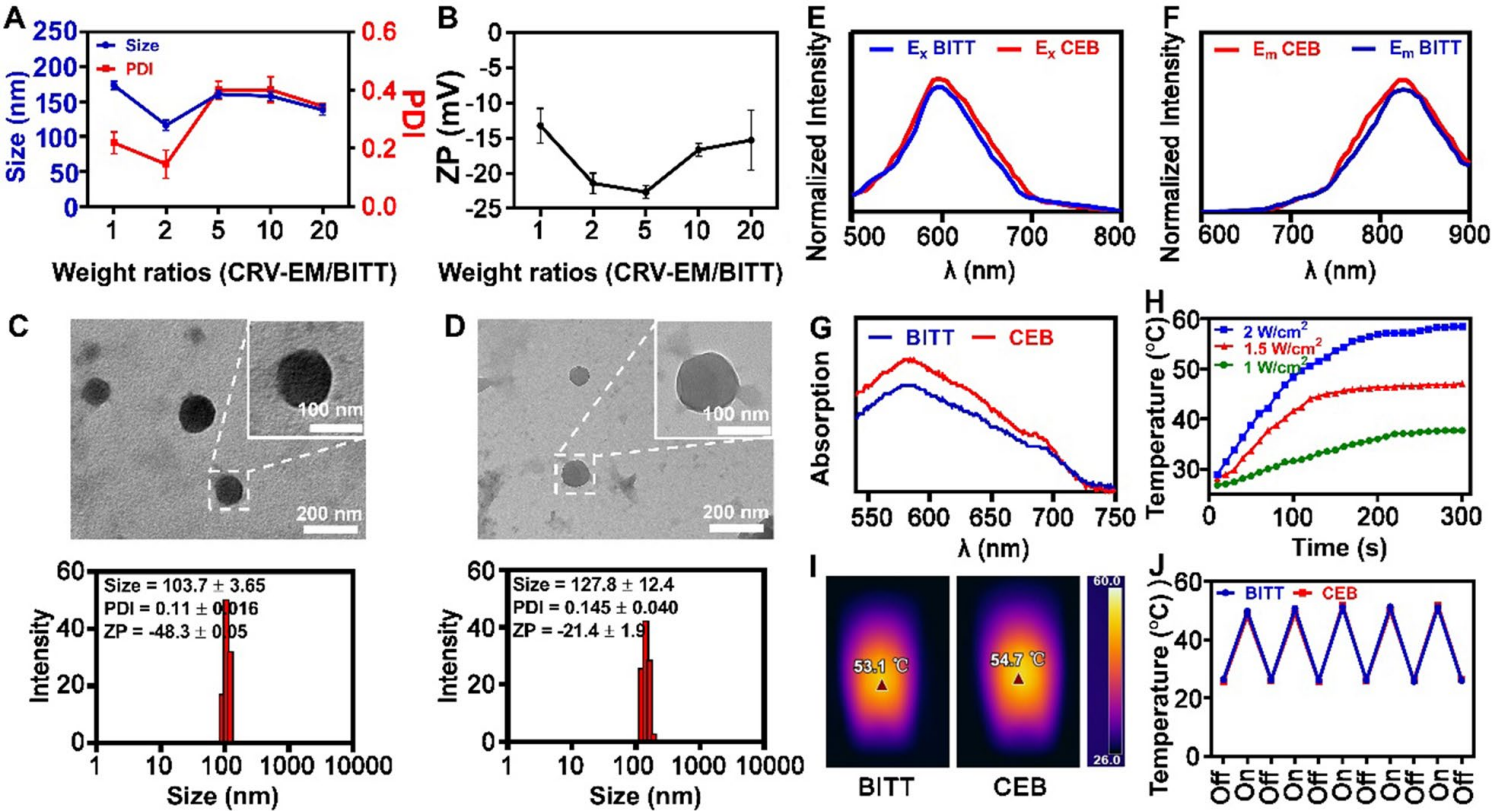
The characterization of BITT-based nanoparticles. **A** The optimized condition to form CEB. **B** Zeta potential of CEB with different formulations. **C** TEM and DLS analysis of BITT. **D** TEM and DLS analysis of CEB. **E** Excitation spectrum of BITT and CEB. Emission wavelength, 820 nm. **F** Emission spectrum of BITT and CEB. Excitation wavelength, 590 nm. **G** The UV spectrum of BITT nanoparticles and CEB. **H** Photothermal conversion of CEB (BITT equivalent to 15 μg/mL) with different laser irradiation power densities. **I** The photothermal effect induced by CEB (BITT equivalent to 15 μg/mL) with a laser irradiation power density of 2 W/cm^2^. **J** Photothermal stability of BITT and CEB upon 660 nm irradiation (2 W/cm^2^, BITT equivalent to 15 μg/mL). CEB, CRV-EM/BITT; ZP, zeta potential

To prepared BITT nanoparticles, NMR analysis was performed to identify the molecular structure (Additional file 1: Fig. S4), which indicated that ^1^H NMR of BITT was consistent to original published studies [18]. BITT powder was dissolved in DMSO, followed by adding the aqueous solution at the weight ratio of 1:9 under water bath ultrasound. BITT showed obvious Tyndall phenomenon in aqueous solution after the container were irradiated by a red-light beam (660 nm) (Additional file 1: Fig. S5A), implying that BITT formed nanoparticles in aqueous solution. In contrast, there is no Tyndall phenomenon in DMSO solution, indicating that BITT is in dissolved state. Furthermore, BITT showed significant fluorescence in the presence of excitation at 660 nm in both DMSO and aqueous solution (Additional file 1: Fig. S5B). We also tested the emission spectrum in the presence of 660 nm irradiation, which indicted that BITT showed significant emission peak at ~ 780 nm (Additional file 1: Fig. S6). To improve the surface functions of BITT nanoparticles, CRV-EM was used to coat BITT (CRV-EM/BITT), and the formulated nanoparticles were designated as CEB. We optimized the preparation of CEB by adjusting CRV-EM ratios to BITT. When the weight ratio of CRV-EM to BITT was 2, the size of nanoparticles attained a minimum status at 127.8 ± 12.4 nm (Fig. 2A) with a zeta potential of ~ − 22 mV (Fig. 2B). The flow cytometry (FACS) analysis also indicated that the higher weight ratio of CRV-EM to BITT (> 2) did not increase the mean fluorescence intensity (MFI) level significantly in the cells (Additional file 1: Fig. S7). Transmission Electron Microscope (TEM) analysis indicated that BITT was spherical with a size of ~ 103.7 ± 3.6 nm (Fig. 2C). The CRV-EM camouflage induced an apparent layer on BITT with a ~ 20 nm increase in size (Fig. 2D), which was confirmed to retain almost the same protein profiles with CRV-EM evidenced by Coomassie blue staining (Additional file 1: Fig. S8). We further analyzed the stability of BITT and CEB within 72 h. BITT and CEB showed good stability since the particle size was less than 200 nm in the period (Additional file 1: Fig. S9). CEB was prepared successfully by the camouflage with CRV-EM to BITT nanoparticles.

### Photothermal and photodynamic performance in vitro

We analyzed the spectrographic properties of CEB. The excitation spectrum indicated that the maximum excitation wavelength of BITT and CEB was 590 nm (Fig. 2E). Correspondingly, the emission spectrum showed that the maximum emission wavelength of BITT and CEB was 820 nm (Fig. 2F). The UV absorption also indicated that the maximum absorption peak was at ~ 590 nm (Fig. 2G). Singlet Oxygen Sensor Green (SOSG) was applied to detect the emergences of ROS. BITT, EB, and CEB in dark showed low level of ROS (characterized by the fluorescence intensity at 525 nm), but obvious increasing the one after exposure to the laser irradiation (Additional file 1: Fig. S10). BITT-base nanoparticles can produce high level of ROS when exposed to laser, implying a good photodynamic performance. The CRV-EM camouflage didn’t affect the fluorescence characteristic of BITT significantly.

To evaluate photothermal performance of BITT-based nanoparticles, the nanoparticles were exposed to a 660 nm laser under different conditions. The heating curve showed that both BITT and CEB possessed excellent photothermal properties. The CEB-induced temperature increase depended on the concentration, power density, and exposure time (Additional file 1: Fig. S11 and Fig. 2H). After exposure to a 660 nm laser (2 W/cm^2^), the solution was quickly heated up to around 50 ℃ within 2 min and rose to ~ 60 °C within 5 min (Fig. 2H). The thermal imaging system confirmed that the thermal effect induced by CEB was similar to the BITT-induced one ( Fig. 2I). An excellent photothermal agent requires the ability to produce constant high temperatures to facilitate repeated photothermal treatments. For the detection of photothermal stability, BITT-based nanoparticles were tested by alternate heating and cooling cycle with irradiation (660 nm, 2 W/cm^2^). The heating curve indicated that each cycle was repeatable with a temperature of over 50 °C (Fig. 2J). Repeated laser exposure induced ignorable changes in the photothermal properties, indicating that BITT-based nanoparticles had excellent photo-heat conversion capacity. We also investigated the influence of the temperature increase of the exosomal coating quality and content, which indicated that there was no obvious difference with or without laser irradiation induced temperature changes (~ 55 °C) by DLS and Zeta potential analysis (Additional file 1: Fig. S12). The data above demonstrated the BITT-based nanoparticles possessed excellent photothermal properties, which was useful in photothermal therapy.

### Optimization of cellular uptake conditions

We optimized the cellular uptake conditions for both LLC cells and M2 macrophages by confocal laser scanning microscopy (CLSM) and FACS analysis. To study the time-dependent cellular uptake, LLC cells were incubated with an equivalent dose of CEB at different time. CLSM analysis showed that the fluorescence intensity within the cells increased with incubation time at an excitation wavelength of 633 nm (Additional file 1: Fig. S13). FACS analysis indicated that BITT-positive cells reached 100% at 6 h, and the further incubation showed no significant changes (Additional file 1: Fig. S13). Furthermore, the dosage-dependent uptake of CEB was determined. CLSM analysis showed that fluorescence intensity within cells increased with the elevation of CEB dosage. FACS analysis indicated that the incubation with CEB (BITT equivalent to 15 μg/mL) for 6 h induced 99.9% of the BITT-positive LLC cells (Additional file 1: Fig. S14). We also evaluated the cellular uptake in M2 macrophages. Similarly, M2 macrophages also showed effective cellular uptake by incubation for 6 h (Additional file 1: Fig. S15) and CEB at a BITT concentration equivalent to 15 μg/mL (Additional file 1: Fig. S16). Thus, the cellular uptake of CEB at a BITT dosage of 15 μg/mL and incubation for 6 h was selected for further experiments.

**Fig. 3:**
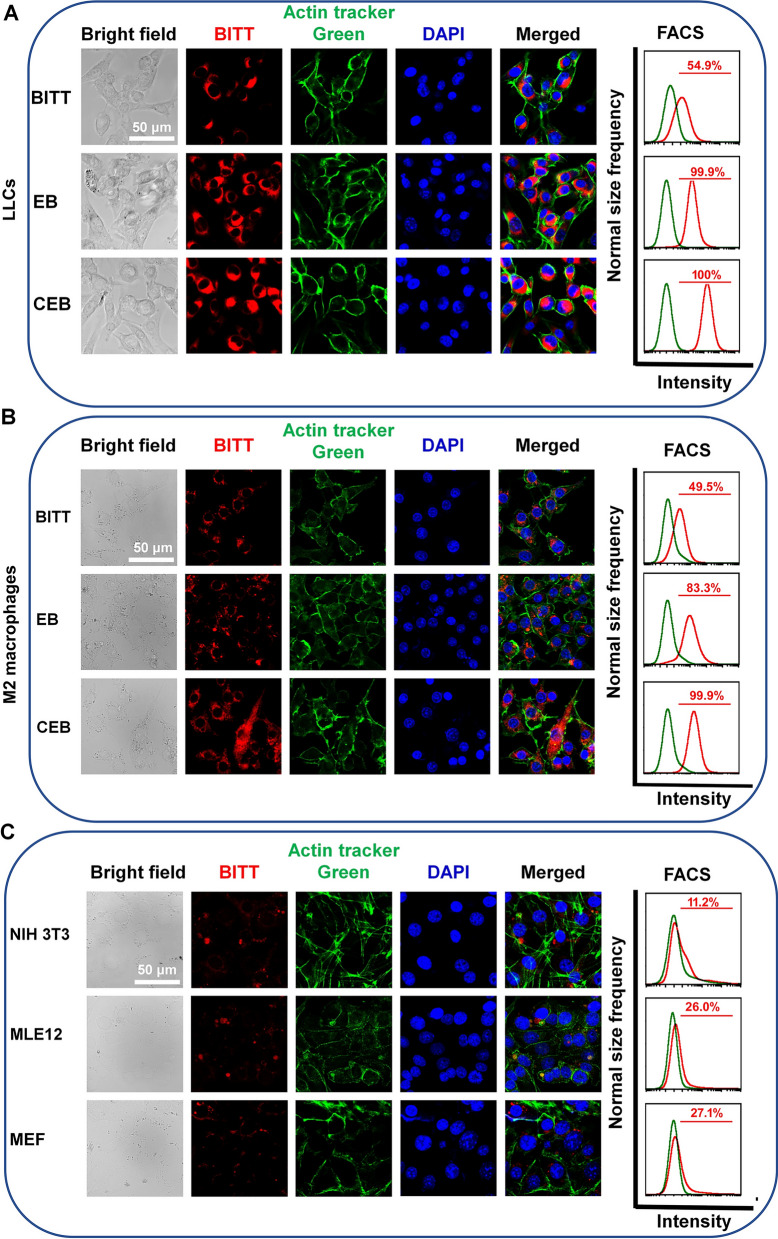
The cellular uptake and cell line specificity. **A** The cellular uptake of different nanoparticles in LLC cells. **B** The cellular uptake of different nanoparticles in M2 macrophages. **C** The cellular uptake of CEB in different cell lines. The cells were treated with CEB at a BITT dosage of 15 μg/mL and incubation for 6 h

To determine the cellular uptake of different formulations, LLC cells or M2 macrophages were treated with BITT, LLC cells exosome membranes decorate BITT nanoparticles (EB), or CEB, respectively. CLSM and FACS analysis was used to test BITT-positive cells. EB and CEB significantly increased the fluorescence intensity within LLC cells compared with naked BITT (Fig. 3A). FACS analysis also indicated that BITT-positive cells were 100% in both EB and CEB-treated cells, compared with 54.9% in BITT-treated ones (Fig. 3A). The evidence suggested that the coating with EM or CRV-EM derived from LLC cells facilitated the cellular uptake by LLC cells. Interestingly, CEB showed the most effective cellular uptake in M2 macrophages compared with BITT or EB. EM coating elevated BITT-positive cells from 49.5 to 80.0%, and CRV-EM coating increased to 99.9% (Fig. 3B). These results suggested that the presence of CRV-EM on CEB increased the cellular uptake in both LLC cells and M2 macrophages due to the homotypic targeting of LLC-derived exosomes and the M2 macrophage-specific peptides. We also evaluated the selectivity of CEB in different cell lines, such as NIH 3T3 cells, MLE12 cells, and MEF. CLSM analysis and FACS analysis indicated that the cellular uptake of CEB in three cell lines was less than 30%, much lower than that of M2 macrophages (Fig. 3C). CEB showed high-level cellular uptake in both LLC cells and M2 macrophages but significantly lower levels in various normal cell lines.

The results indicated that CEB showed a dual-targeting capability to both LLC cells and M2 macrophages, which improved the cellular uptake in both cell lines. On the one hand, CRV peptide-expressed on CEB preferentially bound to M2 TAM by conjugation with retinoid X receptor beta [25]. On the other hand, the CRV-Exosomes derived from LLC cells also targeted LLC cells through a homotypic targeting effect. The selectivity of CEB may contribute to the increase of cellular uptake in both LLC cells and M2 macrophages and the reduction of potential side effects to the normal cells. This property showed great promise for the specific delivery in vivo.

### Phototherapeutic effect of CEB in vitro

**Fig. 4:**
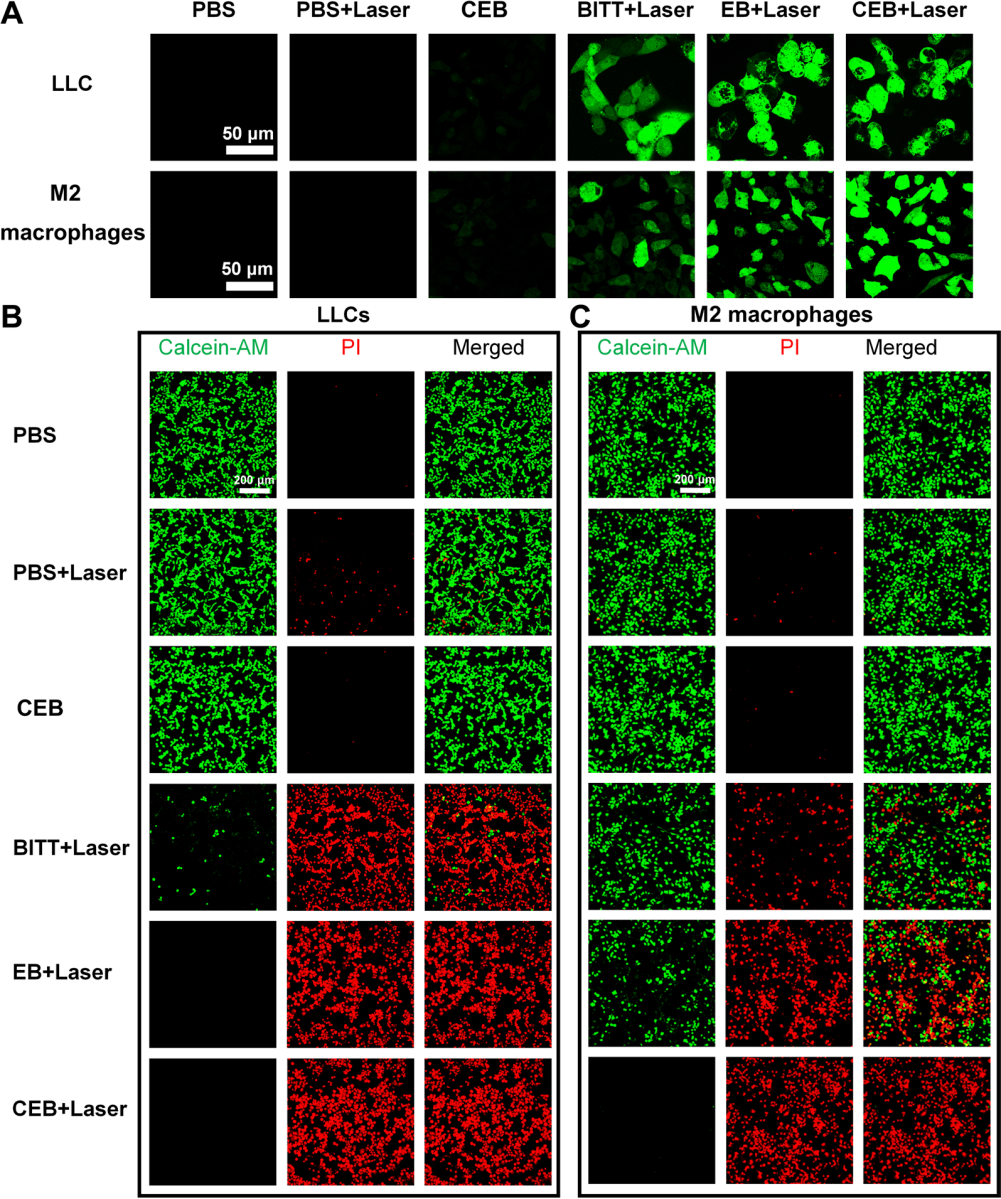
Biological effect induced by BITT-based nanoparticles. **A** ROS level in LLC cells and M2 macrophages induced by different formulations. **B** Live/Dead assay analysis of LLC cells induced by different formulations. **C** Live/Dead assay analysis of M2 macrophages induced by different formulations

We evaluated the photodynamic and photothermal effects induced by different formulations. The intracellular reactive oxygen species (ROS) generation of BITT was evaluated by using DCFH-DA as an indicator. When exposed to the laser, EB and CEB induced a significantly higher level of ROS in both LLC cells and M2 macrophages compared with the one with BITT + Laser (Fig. 4A). The BITT-based nanoparticles without 660 nm laser irradiation did not generate obvious ROS in these cells. The photothermal effect was also monitored with a thermal imaging camera. EB and CEB induced a temperature of ~ 60 °C in both cell lines with the exposure to 660 nm laser irradiation (Additional file 1: Fig. S17). The BITT-based nanoparticles without 660 nm laser irradiation did not induce a significant photothermal effect. The excellent photodynamic and photothermal effect may be due to the increased cellular uptake of EB and CEB, as indicated in the results mentioned above.

To evaluate the in vitro cell viability and phototherapeutic effect, we measured the cell viabilities induced by BITT-based nanoparticles through CCK8 assay, which indicated that BITT, EB and CEB without laser irradiations showed no significant cytotoxicity even with a high BITT concentration at 20 μg/mL (Additional file 1: Fig. S18A and S18B). Upon 660 nm laser irradiation for 5 min, the cell viability significantly decreased with the increase of BITT concentration. CEB + Laser induced the cell inhibition over 80% when BITT concentration exceeded 15 μg/mL (Additional file 1: Fig. S18A and S18B). We also evaluated the CEB functioned in the normal cell lines, such as NIH 3T3, MLE12, and MEF cells with CCK-8 assay. Although CEB induced a 30 ~ 40% cell inhibition in the presence of irradiation, the cell viability was obviously higher than those of LLC and M2 macrophages (Additional file 1: Fig. S19), which might be due to the lower cellular uptake. CEB was efficient in killing the LLC cells and M2 macrophages.

Live/Dead assay was used to assess the viability by using Calcein-AM (green fluorescence for live cells) and propidium iodide (PI, red fluorescence for dead cells) as indicators. LLC cells and M2 macrophages were exposed to different treatments (PBS, PBS + Laser, CEB, BITT + Laser, EB + Laser, and CEB + Laser). BITT + Laser, EB + Laser, and CEB + Laser induced a nearly complete elimination of LLC cells (Fig. 4B). However, the control groups, such as PBS, PBS + Laser, and CEB without laser irradiation, didn’t induce apparent cell death in LLC cells. Similarly, the BITT-based nanoparticles with laser (BITT + Laser, EB + Laser, and CEB + Laser) also caused significant cell death in M2 macrophages. Notably, CEB + Laser induced a much higher cell death level than BITT + Laser, possibly due to the camouflage with CRV-EM enhanced the specific delivery (Fig. 4C). CEB was effective in killing both LLC cells and M2 macrophages in the exposure to laser irradiation.

CEB enabled generating high levels of ROS and heat in the treated cells. In the exposure to the laser, CEB induced significant cell inhibition in vitro, which demonstrated that CEB was useful in photo-mediated therapy.

### Location and inhibition in three-dimensional (3D) tumor spheroids

**Fig. 5:**
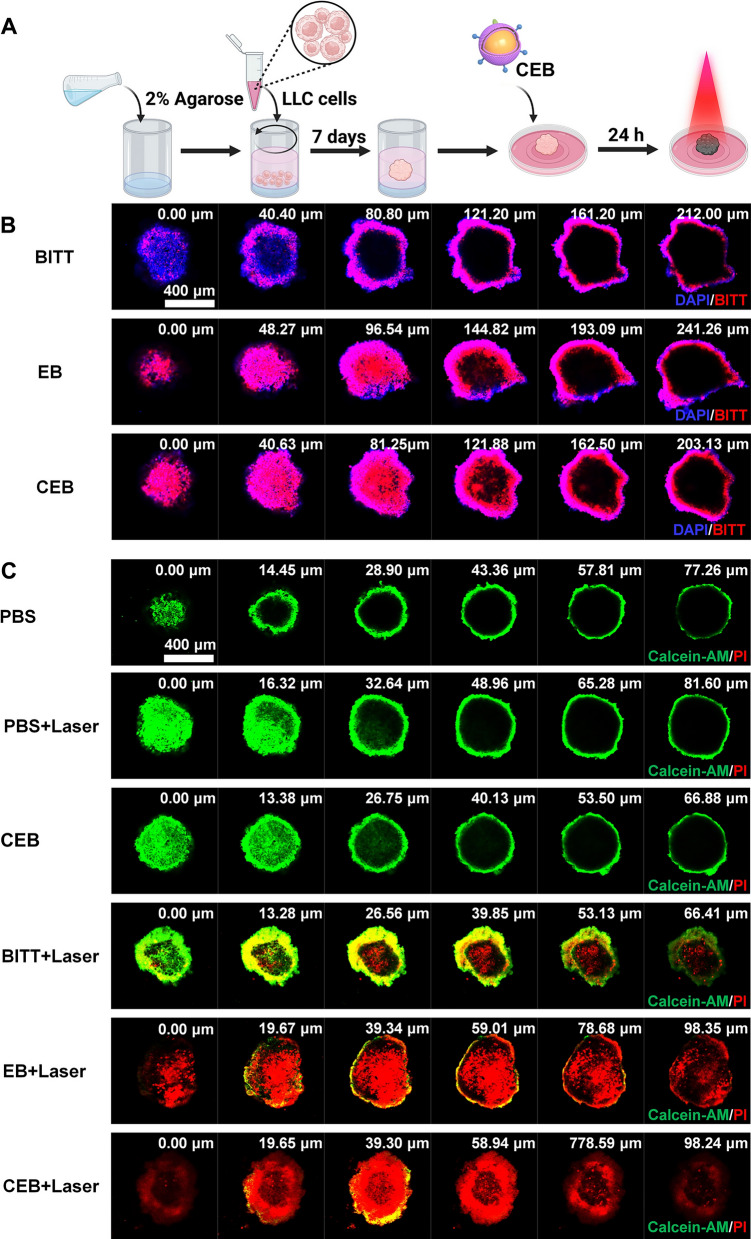
The evaluation of BITT-based nanoparticles in 3D tumor spheroids. **A** Scheme illustration of preparing 3D tumor spheroids. **B** The location of different nanoparticles in 3D tumor spheroids. **C** Live/Dead assay analysis of the 3D tumor spheroids

Encouraged by the excellent performance in the cultured cells in vitro, we prepared 3D tumor spheroids to evaluate the effect of different formulations. We investigated the permeability of different formulations in the 3D tumor spheroids (Fig. 5A). The 3D tumor spheroids were treated with BITT, EB, and CEB for 12 h, and a Z-stack scan was performed on CLSM. The results showed that the red fluorescence of BITT mainly presented on the surface when treated with naked BITT. In contrast, the fluorescence could be observed in the inner layer of tumor spheroids by EB and CEB treatment, implying that the camouflage with EM or CRV-EM derived from LLC cells increased the permeability of BITT (Fig. 5B), which may strengthen the ablation of tumor cells with AIE-based nanoparticles. Furthermore, after the 3D tumor spheroids were cultured with the formulations for 12 h, the cells were exposed to a 660 nm laser (2 W/cm^2^, 5 min). Z-stack scan was acquired to explore the viability of tumor cells in the spheroids by Live/Dead assay. The 3D tumor spheroids that treated with EB + Laser and CEB + Laser showed nearly complete cell death (Fig. 5C), including the interior ones. BITT + Laser induced significant cell death, but the dead cells were mainly located on the superficial layer of the spheroids. In contrast, PBS, PBS + Laser, and CEB without laser irradiation hardly affected the cells. Collectively, CEB enabled to ablate the LLC cells and M2 macrophages effectively. Furthermore, the camouflage with CRV-EM improved the cellular uptake in 3D tumor spheroids, which inspired us to use CEB for in vivo tumor inhibition.

### Biodistribution of CEB

**Fig. 6:**
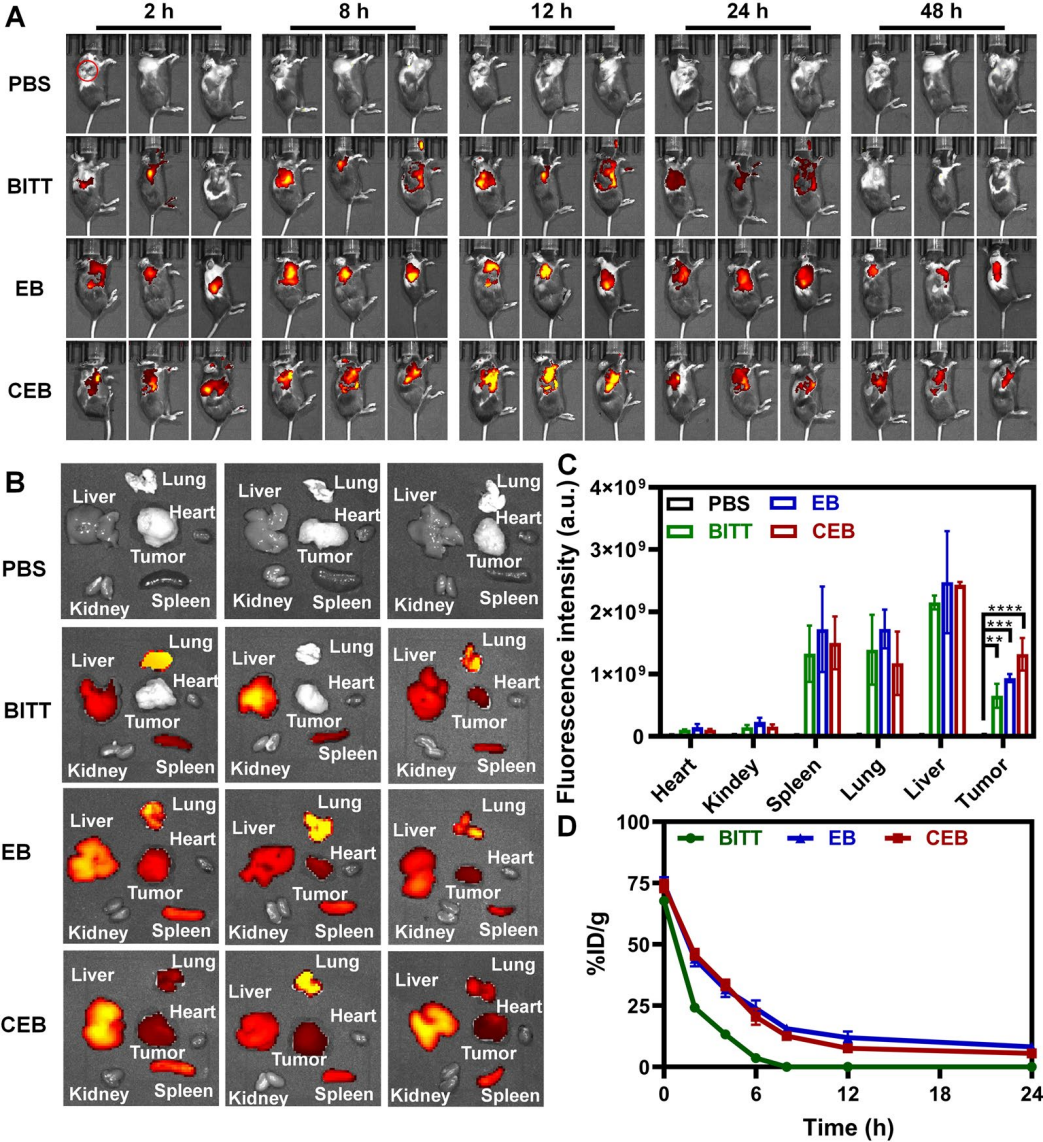
In vivo tracking of BITT-based nanoparticles. **A** The biodistribution of different formulations in vivo. **B** Ex vivo imaging of the organs extracted from the mice administrated with different formulations. **C** The quantitative analysis of the fluorescence in the organs. **D** The circulation lifetime of different formulations. The red circles represent the tumor sites. *p < 0.05, **p < 0.01, and ***p < 0.001; ****, *p* < 0.0001

We used a PerkinElmer IVIS Spectrum In Vivo Imaging System to investigate the distribution of CEB in tumor-bearing mice. To avoid the possible influence of the fluorescence in the liver, the mice were subcutaneously inoculated LLC cells on the right shoulder. After intravenous injection, we monitored the fluorescence signals at 2, 4, 8, 12, 24, and 48 h (Fig. 6A). Interestingly, CEB showed the highest fluorescence intensity in tumor sites at different time points (Fig. 6A). The fluorescence signal in the tumors increased within 4 h and reached a plateau at 8 h. Ex vivo imaging of the organs after 48 h was consistent with the observation from in vivo imaging (Fig. 6B). Quantitative analysis of fluorescence intensity confirmed the high-level accumulations induced by CEB (Fig. 6C). The data demonstrated the superior performance of CEB in tumor-targeted delivery.

The superior material interface endows the nanoparticles with the ability of long circulation lifetime in vivo, improving the efficiency of targeted delivery and therapy [26, 27]. The blood concentration of nanoparticles was measured by fluorescence spectrophotometry. The membrane coating nanoparticles CEB showed a concentration of nanoparticles with over 15% at 8 h after administration (Fig. 6D), which was considerable to the one of EB. However, BITT without membrane coating was immediately cleared from the blood circulation within 8 h, when their concentration was nearly negligible (Fig. 6D). The evidence indicated that the camouflage with EM ameliorated the surface functions, reduced the opsonization by blood proteins and phagocytosis by immune cells, and improved the circulation lifetime [28]. The long circulation lifetime and the enhanced tumor accumulation of CEB ensured efficient photodynamic and photothermal therapy for lung cancer therapy.

### In vivo antitumor effects of CEB

**Fig. 7:**
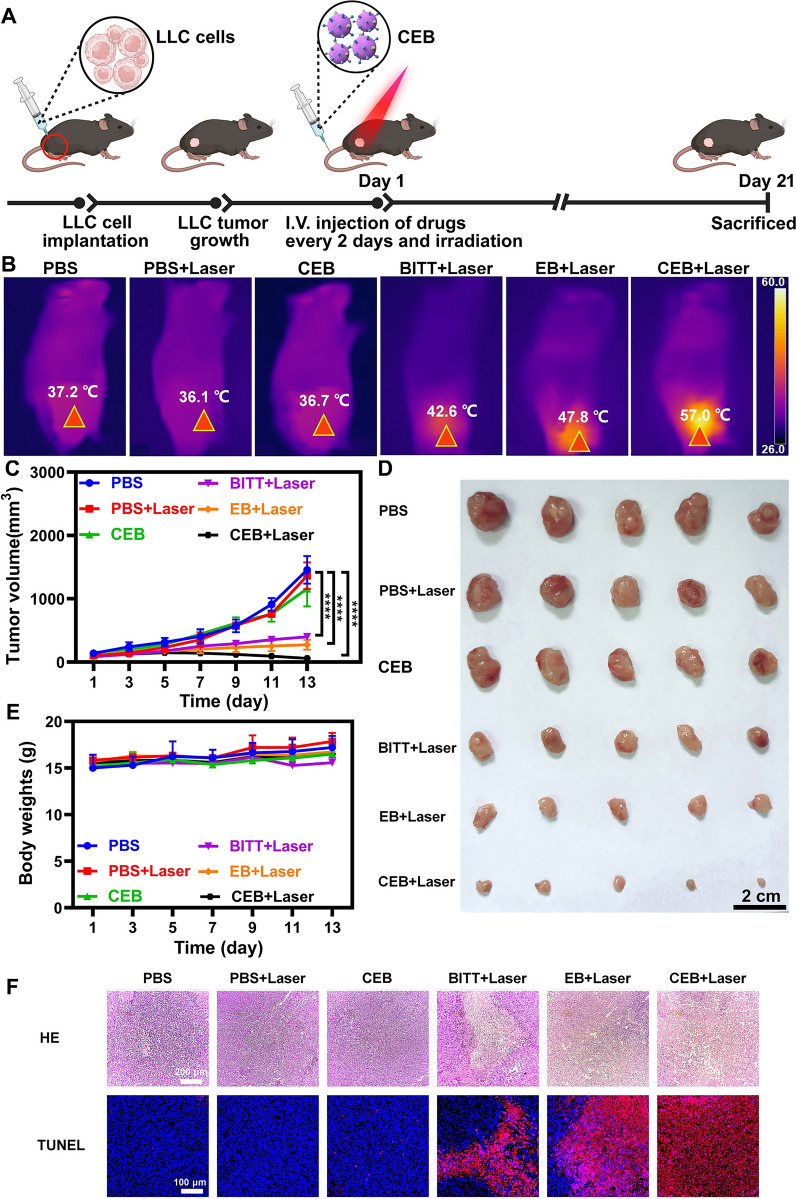
Tumor inhibition induced by BITT-based nanoparticles. **A** Scheme illustration of the drug administration. **B** Thermal imaging after the drug administration. **C** The change of tumor volumes. **D** The images of extracted tumors. **E** The change of body weight. **F** HE and TUNEL analysis of the tumor tissues after the drug administration. *p < 0.05, **p < 0.01, and ***p < 0.001; ****, *p* < 0.0001

We evaluated the effect of CEB on tumor suppression in LLC tumor xenograft mouse models. Mice were randomly assigned to 6 groups and administered with different formulations (PBS, PBS + Laser, CEB, BITT + Laser, EB + Laser, and CEB + Laser). The formulations were administered every 2 days, and the laser irradiation was performed after the drug administration for 8 h based on the high retention in tumors (Fig. 6A and Fig. 7A). Thermal imaging of the tumors indicated that the treatment with CEB + Laser induced a temperature of ~ 60 °C (Fig. 7B). In contrast, the control groups, such as PBS, PBS + Laser, CEB, and BITT + Laser, showed ~ 15–30 °C lower, compared with the treatment with CEB + Laser (Fig. 7B), implying the improved accumulation of BITT induced a stronger thermal effect. EB + Laser induced a temperature of ~ 47 °C but was also significantly lower than CEB + Laser treated ones. Particularly, CEB + Laser led to the most effective reduction in the tumors, which was ~ 1/20 of the volume of the PBS or PBS + Laser treated ones (Fig. 7C). Compared with PBS, BITT without laser-induced no apparent change in tumor growth as indicated by measured tumor volume. BITT under laser irradiation mildly reduced the tumor size (Fig. 7C), possibly due to the low accumulation of BITT with the non-specific distribution. Although EB + Laser also showed preferable tumor size reduction (~ 1/5 of the ones of PBS treated mice), the therapeutic effect was inferior to CEB + Laser -treated mice. The extracted tumors confirmed the therapeutic effect induced by different formulations (Fig. 7D), which demonstrated the CEB + Laser was the most effective in the suppression of the tumor growth. We monitored the bodyweights of the mice and found no significant weight loss during the therapeutic process, implying the treatment with CEB + Laser was a safe approach for tumor therapy (Fig. 7E). Hematoxylin–eosin (HE) staining indicated that CEB + Laser induced significant necrosis, evidenced by the reduced stained nucleus (Fig. 7F). TdT-mediated dUTP Nick-End Labeling (TUNEL) was used to evaluate the apoptosis of tumor tissues. CEB + Laser showed the most apparent apoptosis compared with the control groups, such as PBS, PBS + Laser, CEB, and BITT + Laser (Fig. 7F). The quantitative analysis also confirmed that CEB + Laser induced a high level of apoptosis (Additional file 1: Fig. S20), which was much more effective than other treatments. Although the heating area was larger than the tumor sites at present, the laser spot would be adjustable in the future applications. Based on the above results, the dual targeting CEB showed excellent antitumor effects. CEB targeted both lung cancer cells and M2 TAMs, followed by elimination of both cells in the presence of laser, potentially attributed to the efficient photodynamic and photothermal effect.

### The effect on tumor microenvironmental remodeling

**Fig. 8:**
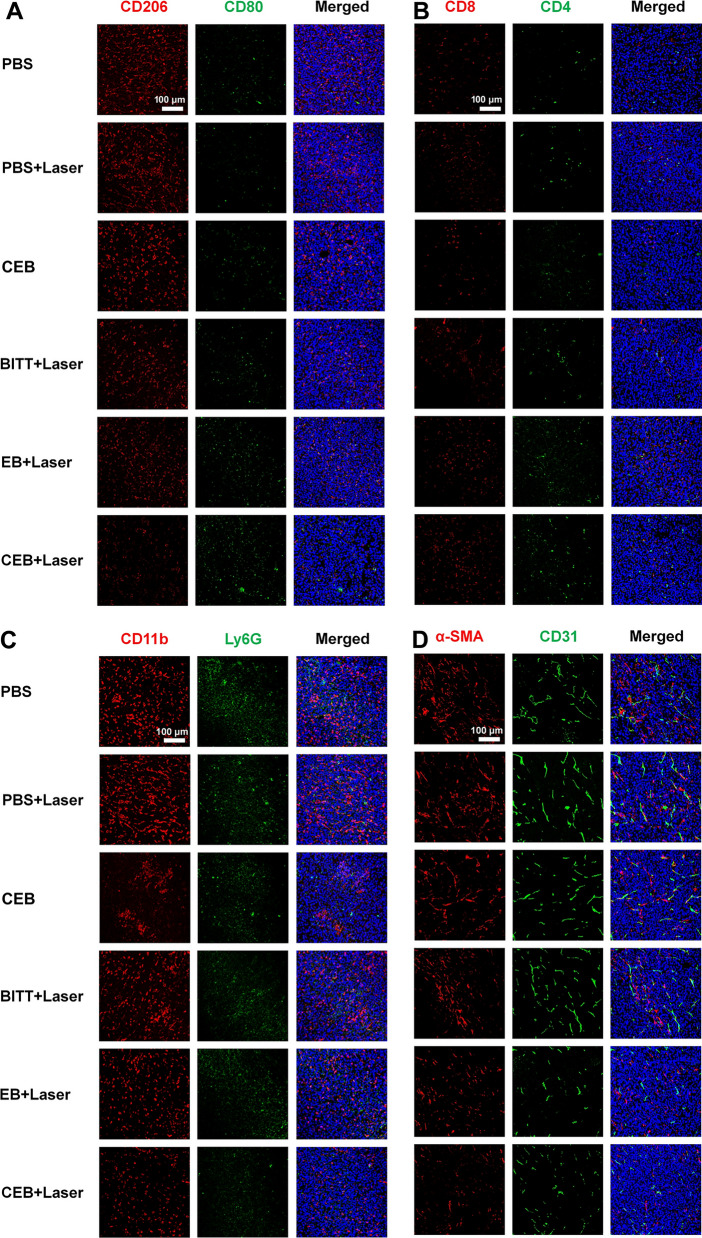
The immunofluorescence analysis of the tumor tissues after the treatment with BITT-based nanoparticles. **A** The identification of macrophages. **B** The identification of T cells. **C** The identification of MDSCs. **D** The identification of blood vessels

To further study the mechanisms underlying the antitumor effects of CEB, we analyzed the amounts of immune cells, such as M1 and M2 TAMs, T cells, and MDSCs in the tumor microenvironment by immunofluorescence analysis. F4/80^+^CD80^+^ or F4/80^+^CD206^+^ were considered as the markers of M1 or M2 TAMs, respectively. CLSM and FACS analysis indicated that CEB + Laser induced the most significant changes in the cell population, in which M1 TAMs increased by ~ 10% and M2 TAMs decreased to about 1/2 of the PBS treated ones (Fig. 8A, Additional file 1: Fig. S21A, B). The ratio of M1 to M2 TAMs showed a ~ twofold increase compared with PBS-treated ones (Additional file 1: Fig. S21C). However, the effect induced by BITT + Laser, EB + Laser, and CEB without laser irradiation was much less compared with the treatment with CEB + Laser (Fig. 8A, Additional file 1: Fig. S21A, B, and C). The changes in TAMs profiles were correlated with the increases in the CD3e^+^CD4^+^ and CD3e^+^CD8^+^ T cell infiltration in tumors, and the effects of CEB + Laser were most evident (Fig. 8B, Additional file 1: Fig. S21D, E). Furthermore, the infiltration of immunosuppressive MDSCs (CD45^+^CD11b^+^Gr1^+^) was reduced in half after the treatment with CEB + Laser (Fig. 8C and Additional file 1: Fig. S21F). Interestingly, we also studied the effects of angiogenesis. CEB led to a significant decrease of α-SMA and CD31 in the presence of laser irradiation (Fig. 8D), implying that the AIE-induced therapeutic effect inhibited the tumor angiogenesis. These results indicated that CEB with laser irradiation remodeled the tumor environment, characterized by increased M1 TAMs, CD8^+^, and CD4^+^ T cells and a decrease in M2 TAMs and MDSCs. This gene engineering generated CRV-EM endowed the AIE-based nanoparticles with dual targeting capability and targeted both cancer cells and M2 TAMs. This effectively inhibited the tumor growth by killing cancer cells and M2 TAMs, followed by remodeling the tumor environment. Our strategy opened a new avenue for effective cancer therapy with AIE-mediated immunotherapy.

### Biosafety assessment

**Fig. 9:**
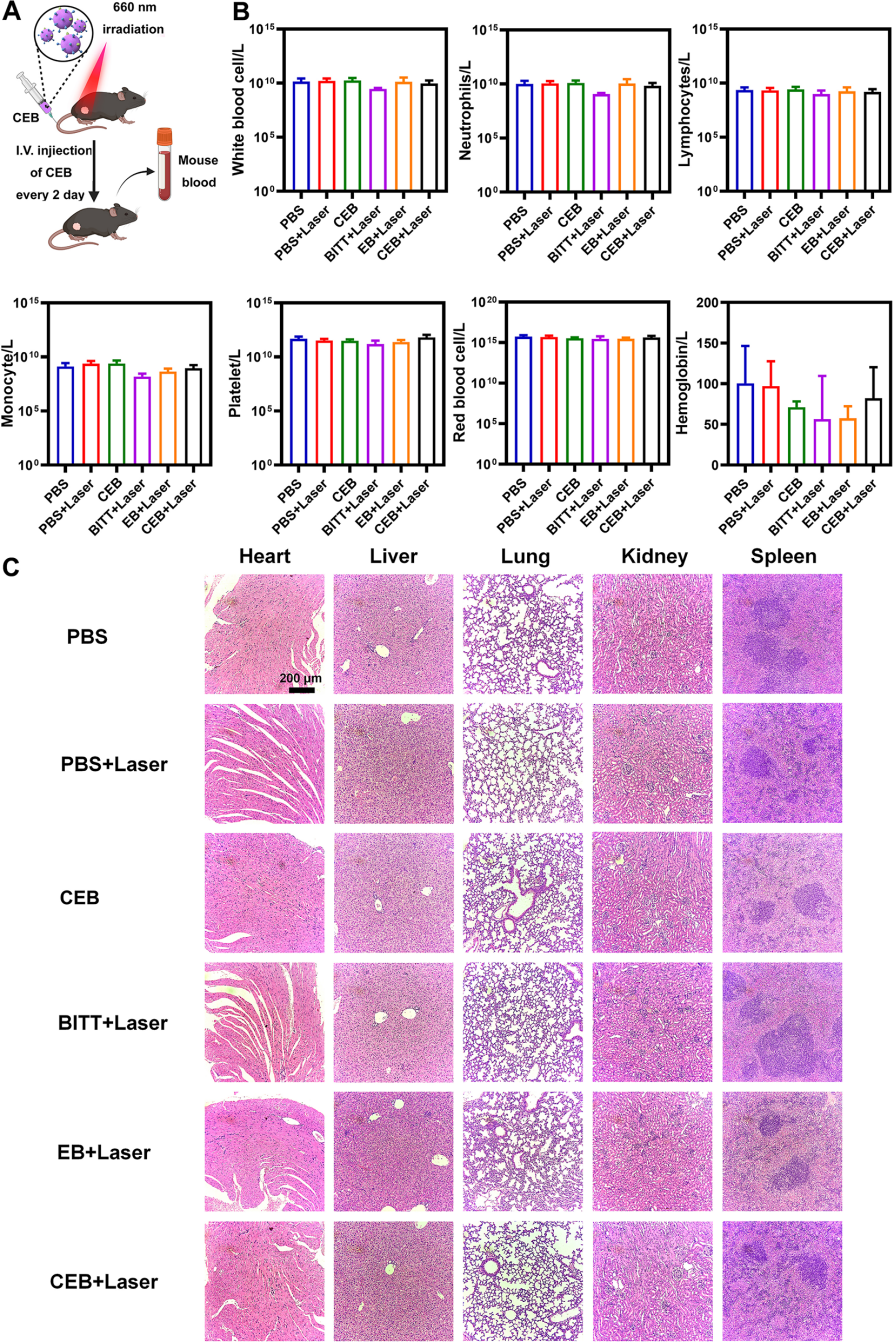
The biosafety of BITT-based nanoparticles. **A** Scheme illustration of the blood collection after drug administration. **B** The routine analysis of blood. **C** HE staining the major organs

To evaluate the biosafety of CEB-induced therapy, the blood routine and pathological analysis were performed. After the complement of drug administration on Day 13, the mouse blood was collected (illustrated in Fig. 9A). The blood routine analysis indicated that the above treatments showed an ignorable effect on the parameters, such as white blood, neutrophils, lymphocytes, monocytes, platelets, red blood cells, and hemoglobin (Fig. 9B). Furthermore, HE staining indicated no significant histological changes in the major organs such as the heart, liver, lung, kidneys, and spleen after the treatment with the PBS, PBS + Laser, CEB, BITT + Laser, EB + Laser, and CEB + Laser (Fig. 9C), respectively. The data was consistent with previous work, which indicated that BITT aggregates released from the carrier after intravenous (i.v.) injection excreting through the biliary system into the intestine [19], which might be metabolized within days without significant side effects. The evidence indicated that the AIE-based cancer therapy showed good biosafety cancer therapy, which was critically important for its potential applications in the future.

## Conclusion

In conclusion, we developed a novel style of bioinspired AIE aggregates (CEB) camouflaged with the engineered exosome-derived membranes. CEB enabled to targeted both lung cancer cells and TAMs through homotypic targeting and TAM-specific peptide, respectively, and thus inducing efficient photodynamic and photothermal therapy in vitro and in vivo. Moreover, the laser irradiated CEB reversed the tumor-promoting microenvironment into tumor-suppressive one, and achieved an efficient lung cancer therapy. Although the penetration depth of this type of photosensitizer is usually limited to millimeters at present, the laser can be conducted by optical fiber percutaneously or bronchi interventionally to the lung. This type of bioinspired AIE aggregates showed great potential in photo-mediated immunotherapy for lung cancer therapy.

## Supplementary Information

**Additional file 1: Figure S1.** Scheme illustration of CRV-encoded plasmid. **Figure S2.** Characterizations of CRV-Exosomes. **A** TEM analysis of CRV-Exosomes. **B** Nano-FCM analysis of CRV-Exosomes. **Figure S3.** WB analysis of the FLAG, Lamp2b, ALIX and HSP70 proteins derived from cells membrane and exosome membranes. LLCM, LLC cell membranes; EM LLC exosomes membranes; CRV-LLCM, CRV overexpress LLC cell membranes; CEV-EM, CRV overexpress LLC exosomes membranes. **Figure S4.** ^1^H NMR of BITT. ^1^H NMR (500 MHz, Chloroform-d) δ 8.50 (s, 1H), 8.36 (d, J=15.2 Hz, 1H), 8.20-8.02 (m, 3H), 7.83-7.70 (m, 3H), 7.63 (d, J=7.5 Hz, 1H), 7.57 (d, J=8.3 Hz, 2H), 7.48 (s, 1H), 7.33 (t, J=7.7 Hz, 4H), 7.21-7.11 (m, 6H), 7.05 (d, J=8.3 Hz, 2H), 5.05 (s, 2H), 3.19 (s, 2H), 2.50 (s, 2H), 2.02 (s, 6H). **Figure S5.** The physical properties of BITT in different solutions. **A** The verification of Tyndall phenomenon. **B** Fluorescence imaging. **Figure S6** The emission spectrum in the presence of 660 nm irradiation. **Figure S7.** Mean fluorescence intensity (MFI) of the cells treated with CEB prepared with different weight ratios of CRV-EM to BITT. **Figure S8.** SDS-PAGE analysis of the retained proteins on the CEV-EM and CEB. **Figure S9.** Stabilities of nanoparticles in PBS for 72 h. **Figure S10. **ROS level induced by the BITT-based nanoparticles. **Figure S11. **Photothermal conversion of CEB in different concentrations under 660 nm laser irradiation at the intensity of 2W/cm^2^. **Figure S12. **Size and zeta potential of the nanoparticles with or without laser irradiation. **Figure S13.** Cellular uptake of CEB in LLC cells incubated for different time. **Figure S14.** Cellular uptake of different concentrations CEB in LLC cells. **Figure S15.** Cellular uptake of CEB in M2 macrophages incubated for different time. **Figure S16.** Cellular uptake of different concentrations CEB in M2 macrophages. **Figure S17.** Photothermal effect induced by different formulations in cells. **Figure S18.** CCK8 analysis of cell viability. **A** LLC cells viability induced by BITT-based nanoparticles with/without irradiation. **B** M2 macrophages viability induced by BITT-based nanoparticles with/without irradiation. **Figure S19.** Cell viability of different cells induced by CEB. Different cell lines including LLC, M2 macrophages, NIH 3T3, MLE12, and MEF were treated with CEB at a BITT concentration of 15 μg/mL and exposed to the 660 nm irradiation for 5 min. CCK-8 assay was performed to test the cell viability. **Figure S20.** The quantitative analysis of the apoptosis in the tumors. **Figure S21.** Quantitatively analysis of the immune cells after the treatment with different formulations. **A** M1 macrophages. **B** M2 macrophages. **C** The ratio of M1 to M2 macrophages. **D** CD8+ T cells. **E** CD4+ T cells. **F** MDSC cells.

## Data Availability

The datasets used and/or analyzed during the current study are available from the corresponding author on reasonable request.

## Abbreviations

CEB: CRV-engineered lung cancer cell-derived exosomes membranes/BITT
LLC: Lewis lung carcinoma
HEK-293T: Human embryonic kidney cells
NIH 3T3: Mouse embryo fibroblast cells
MLE12 cells: Mouse lung epithelial cells
MEF: Mouse embryonic fibroblast
DMEM: Dulbecco’s modified eagle medium
CLSM: Confocal laser scanning microscope
FACS: Fluorescence activated cell sorting

## References

[1] Herbst RS, Morgensztern D, Boshoff C. The biology and management of non-small cell lung cancer. Nature. 2018;553:446–54. 29364287 10.1038/nature25183

[2] Yang LY, Lin YS, Zhang J, Huang JH, Qin AP, Miao YL, et al. Biomimetic metal-organic frameworks navigated biological bombs for efficient lung cancer therapy. J Colloid Interface Sci. 2022;625:532–43. 35749848 10.1016/j.jcis.2022.06.008

[3] Quail DF, Joyce JA. Microenvironmental regulation of tumor progression and metastasis. Nat Med. 2013;19:1423–37. 24202395 10.1038/nm.3394 PMC3954707

[4] Parayath NN, Parikh A, Amiji MM. Repolarization of tumor-associated macrophages in a genetically engineered nonsmall cell lung cancer model by intraperitoneal administration of hyaluronic acid-based nanoparticles encapsulating microrna-125b. Nano Lett. 2018;18:3571–9. 29722542 10.1021/acs.nanolett.8b00689

[5] Xiang XN, Wang JG, Lu D, Xu X. Targeting tumor-associated macrophages to synergize tumor immunotherapy. Signal Transduct Target Ther. 2021;6:75. 33619259 10.1038/s41392-021-00484-9 PMC7900181

[6] Lin YX, Xu JX, Lan HY. Tumor-associated macrophages in tumor metastasis: biological roles and clinical therapeutic applications. J Hematol Oncol. 2019;12:76. 31300030 10.1186/s13045-019-0760-3 PMC6626377

[7] Whiteside TL. The tumor microenvironment and its role in promoting tumor growth. Oncogene. 2008;27:5904–12. 18836471 10.1038/onc.2008.271 PMC3689267

[8] Li M, Li MM, Yang YL, Liu YK, Xie HB, Yu QW, et al. Remodeling tumor immune microenvironment via targeted blockade of PI3K-gamma and CSF-1/CSF-1R pathways in tumor associated macrophages for pancreatic cancer therapy. J Control Release. 2020;321:23–35. 32035193 10.1016/j.jconrel.2020.02.011

[9] Bart VMT, Pickering RJ, Taylor PR, Ipseiz N. Macrophage reprogramming for therapy. Immunology. 2021;163:128–44. 33368269 10.1111/imm.13300 PMC8114216

[10] Gajewski TF, Schreiber H, Fu YX. Innate and adaptive immune cells in the tumor microenvironment. Nat Immunol. 2013;14:1014–22. 24048123 10.1038/ni.2703 PMC4118725

[11] He LZ, Nie TQ, Xia XJ, Liu T, Huang YY, Wang XJ, et al. Designing bioinspired 2D MoSe2 nanosheet for efficient photothermal-triggered cancer immunotherapy with reprogramming tumor-associated macrophages. Adv Funct Mater. 2019;29:1901240.

[12] Shi CR, Liu T, Guo ZD, Zhuang RQ, Zhang XZ, Chen XY. Reprogramming tumor-associated macrophages by nanoparticle-based reactive oxygen species photogeneration. Nano Lett. 2018;18:7330–42. 30339753 10.1021/acs.nanolett.8b03568

[13] Mei J, Leung NL, Kwok RT, Lam JW, Tang BZ. Aggregation-induced emission: together we shine, united we soar! Chem Rev. 2015;115:11718–940. 26492387 10.1021/acs.chemrev.5b00263

[14] Li Y, Tang RB, Liu XY, Gong JY, Zhao ZJ, Sheng ZH, et al. Bright aggregation-induced emission nanoparticles for two-photon imaging and localized compound therapy of cancers. ACS Nano. 2020;14:16840–53. 33197171 10.1021/acsnano.0c05610

[15] Xu XL, Deng GJ, Sun ZH, Luo Y, Liu JK, Yu XH, et al. A biomimetic aggregation-induced emission photosensitizer with antigen-presenting and hitchhiking function for lipid droplet targeted photodynamic immunotherapy. Adv Mater. 2021;33: e2102322. 34247428 10.1002/adma.202102322

[16] Chen XH, Gao HQ, Deng YY, Jin Q, Ji J, Ding D. Supramolecular aggregation-induced emission nanodots with programmed tumor microenvironment responsiveness for image-guided orthotopic pancreatic cancer therapy. ACS Nano. 2020;14:5121–34. 32283914 10.1021/acsnano.0c02197

[17] Wang YB, Wu WB, Mao D, Teh C, Wang B, Liu B. Metal–organic framework assisted and tumor microenvironment modulated synergistic image-guided photo-chemo therapy. Adv Funct Mater. 2020;30:2002431.

[18] Zhu W, Kang MM, Wu Q, Zhang ZJ, Wu Y, Li CB, et al. Zwitterionic aiegens: rational molecular design for NIR-II fluorescence imaging-guided synergistic phototherapy. Adv Funct Mater. 2021;31:2007026.

[19] Ding KK, Wang LR, Zhu JM, He D, Huang YH, Zhang WJ, et al. Photo-enhanced chemotherapy performance in bladder cancer treatment via albumin coated aie aggregates. ACS Nano. 2022;16:7535–46. 35413177 10.1021/acsnano.1c10770

[20] Kalluri R, LeBleu VS. The biology, function, and biomedical applications of exosomes. Science. 2020. 10.1126/science.aau6977. 32029601 10.1126/science.aau6977 PMC7717626

[21] Li H, Li SP, Lin YS, Chen S, Yang LY, Huang X, et al. Artificial exosomes mediated spatiotemporal-resolved and targeted delivery of epigenetic inhibitors. J Nanobiotechnology. 2021;19:364. 34789273 10.1186/s12951-021-01107-9 PMC8597284

[22] Liu C, Zhang W, Li YK, Chang JQ, Tian F, Zhao FH, et al. Microfluidic sonication to assemble exosome membrane-coated nanoparticles for immune evasion-mediated targeting. Nano Lett. 2019;19:7836–44. 31597431 10.1021/acs.nanolett.9b02841

[23] Alvarez-Erviti L, Seow Y, Yin H, Betts C, Lakhal S, Wood MJ. Delivery of sirna to the mouse brain by systemic injection of targeted exosomes. Nat Biotechnol. 2011;29:341–5. 21423189 10.1038/nbt.1807

[24] Tian YH, Li SP, Song J, Ji TJ, Zhu MT, Anderson GJ, et al. A doxorubicin delivery platform using engineered natural membrane vesicle exosomes for targeted tumor therapy. Biomaterials. 2014;35:2383–90. 24345736 10.1016/j.biomaterials.2013.11.083

[25] Tang T, Wei YS, Kang JY, She ZG, Kim D, Sailor MJ, et al. Tumor-specific macrophage targeting through recognition of retinoid X receptor beta. J Control Release. 2019;301:42–53. 30871996 10.1016/j.jconrel.2019.03.009 PMC6500479

[26] Li H, Peng QS, Yang LY, Lin YS, Chen S, Qin YY, et al. High-performance dual combination therapy for cancer treatment with hybrid membrane-camouflaged mesoporous silica gold nanorods. ACS Appl Mater Interfaces. 2020;12:57732–45. 33326211 10.1021/acsami.0c18287

[27] Zhang YF, Yang LY, Wang H, Huang JH, Lin YS, Chen S, et al. Bioinspired metal–organic frameworks mediated efficient delivery of sirna for cancer therapy. Chem Eng J. 2021;426:131926.

[28] Hu CM, Fang RH, Wang KC, Luk BT, Thamphiwatana S, Dehaini D, et al. Nanoparticle biointerfacing by platelet membrane cloaking. Nature. 2015;526:118–21. 26374997 10.1038/nature15373 PMC4871317

