# Correction: “Two birds with one stone” strategy for the lung cancer therapy with bioinspired AIE aggregates

**DOI:** 10.1186/s12951-025-03981-z

**Published:** 2026-01-20

**Authors:** Yinshan Lin, Mengmeng Yi, Xiaoling Guan, Enen Chen, Langyu Yang, Songpei Li, Ying Li, Lingmin Zhang

**Affiliations:** 1 Guangzhou Municipal and Guangdong Provincial Key Laboratory of Molecular Target & Clinical Pharmacology, The NMPA and State Key Laboratory of Respiratory Disease, School of Pharmaceutical Sciences and the Fifth Affiliated Hospital, Guangzhou Medical University, Guangzhou, 511436 China; 2 Key Laboratory of Tropical and Subtropical Fishery Resources Application and Cultivation, Ministry of Agriculture and Rural Affairs; Guangdong Provincial Key Laboratory of Aquatic Animal Immunology and Sustainable Aquaculture Pearl River Fisheries Research Institute, Chinese Academy of Fishery Sciences, Guangzhou, 510380 China

**Correction: Journal of Nanobiotechnology (2023) 21:49**

10.1186/s12951-023-01799-1

In Fig. 9c, the hematoxylin-eosin staining image for the kidney tissue in the CEB + Laser group was inadvertently duplicated as the image for the CEB group. The error occurred during figure assembly and does not affect the authenticity of the experimental data or the conclusions of the study.

The correct hematoxylin-eosin staining image for the CEB group in Fig. 9C has now been updated in the correction.

For completeness and transparency, the old incorrect and correct versions are displayed below.

**Incorrect** **Figure** **9**:

**Fig. 9:**
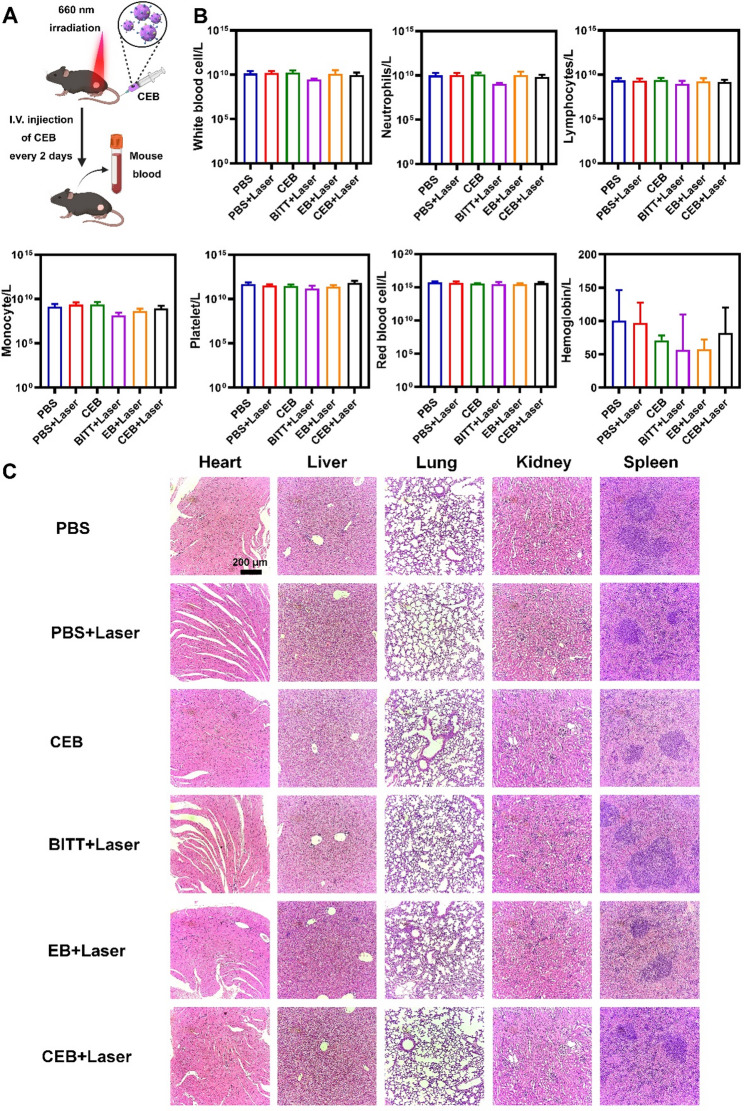
The biosafety of BITT-based nanoparticles. **A** Scheme illustration of the blood collection after drug administration. **B** The routine analysis of blood. **C** HE staining the major organs

**Correct** **Figure** **9**:

**Fig. 9:**
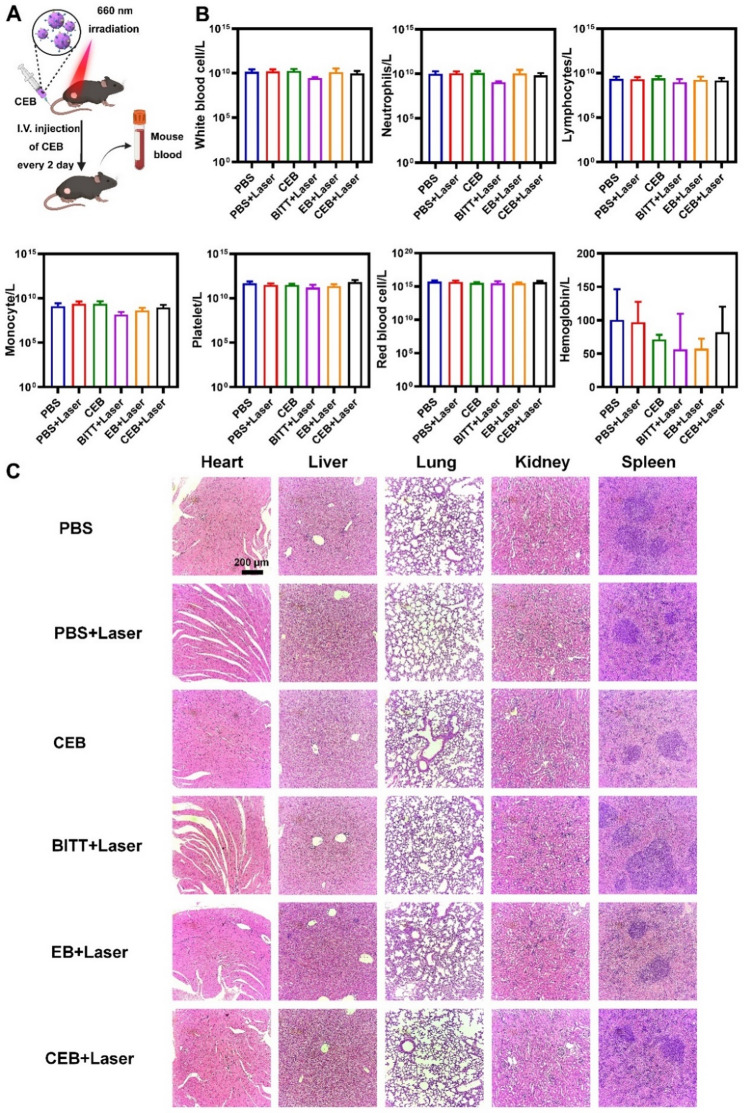
The biosafety of BITT-based nanoparticles. A Scheme illustration of the blood collection after drug administration. B The routine analysis of blood. C HE staining the major organs

The original article has been corrected.

