# Supplementary material for: “Two birds with one stone” strategy for the lung cancer therapy with bioinspired AIE aggregates
Source: J Nanobiotechnology. 2023 Feb 9;21:49. doi: 10.1186/s12951-023-01799-1 (PMC9912660; doi:10.1186/s12951-023-01799-1)
Supplement: Supplementary file 1 — Additional file 1: Figure S1. Scheme illustration of CRV-encoded plasmid. Figure S2. Characterizations of CRV-Exosomes. A TEM analysis of CRV-Exosomes. B Nano-FCM analysis of CRV-Exosomes. Figure S3. WB analysis of the FLAG, Lamp2b, ALIX and HSP70 proteins derived from cells membrane and exosome membranes. LLCM, LLC cell membranes; EM LLC exosomes membranes; CRV-LLCM, CRV overexpress LLC cell membranes; CEV-EM, CRV overexpress LLC exosomes membranes. Figure S4. 1H NMR of BITT. 1H NMR (500 MHz, Chloroform-d) δ 8.50 (s, 1H), 8.36 (d, J=15.2 Hz, 1H), 8.20-8.02 (m, 3H), 7.83-7.70 (m, 3H), 7.63 (d, J=7.5 Hz, 1H), 7.57 (d, J=8.3 Hz, 2H), 7.48 (s, 1H), 7.33 (t, J=7.7 Hz, 4H), 7.21-7.11 (m, 6H), 7.05 (d, J=8.3 Hz, 2H), 5.05 (s, 2H), 3.19 (s, 2H), 2.50 (s, 2H), 2.02 (s, 6H). Figure S5. The physical properties of BITT in different solutions. A The verification of Tyndall phenomenon. B Fluorescence imaging. Figure S6 The emission spectrum in the presence of 660 nm irradiation. Figure S7. Mean fluorescence intensity (MFI) of the cells treated with CEB prepared with different weight ratios of CRV-EM to BITT. Figure S8. SDS-PAGE analysis of the retained proteins on the CEV-EM and CEB. Figure S9. Stabilities of nanoparticles in PBS for 72 h. Figure S10. ROS level induced by the BITT-based nanoparticles. Figure S11. Photothermal conversion of CEB in different concentrations under 660 nm laser irradiation at the intensity of 2W/cm2. Figure S12. Size and zeta potential of the nanoparticles with or without laser irradiation. Figure S13. Cellular uptake of CEB in LLC cells incubated for different time. Figure S14. Cellular uptake of different concentrations CEB in LLC cells. Figure S15. Cellular uptake of CEB in M2 macrophages incubated for different time. Figure S16. Cellular uptake of different concentrations CEB in M2 macrophages. Figure S17. Photothermal effect induced by different formulations in cells. Figure S18. CCK8 analysis of cell viability. A LLC cells viability induced by [file 12951_2023_1799_MOESM1_ESM.docx]

**Additional file information**

**
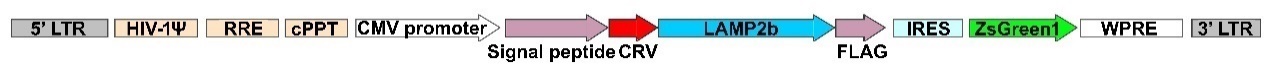
**

**Fig. S1** Scheme illustration of CRV-encoded plasmid.

**
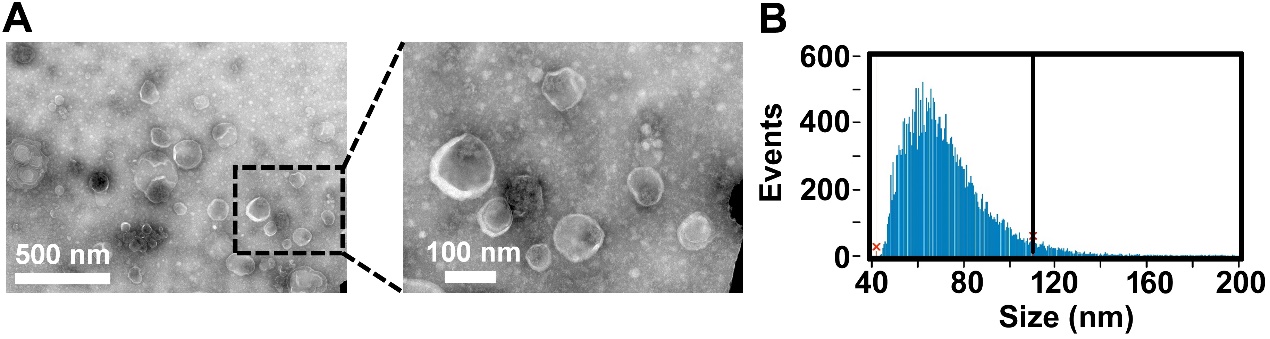
**

**Fig. S2** Characterizations of CRV-Exosomes. **A** TEM analysis of CRV-Exosomes. **B** Nano-FCM analysis of CRV-Exosomes.

**
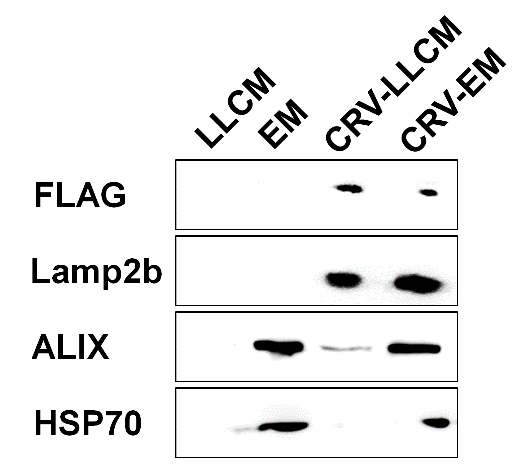
**

**Fig. S3** WB analysis of the FLAG, Lamp2b, ALIX and HSP70 proteins derived from cells membrane and exosome membranes. LLCM, LLC cell membranes; EM LLC exosomes membranes; CRV-LLCM, CRV overexpress LLC cell membranes; CEV-EM, CRV overexpress LLC exosomes membranes.

^
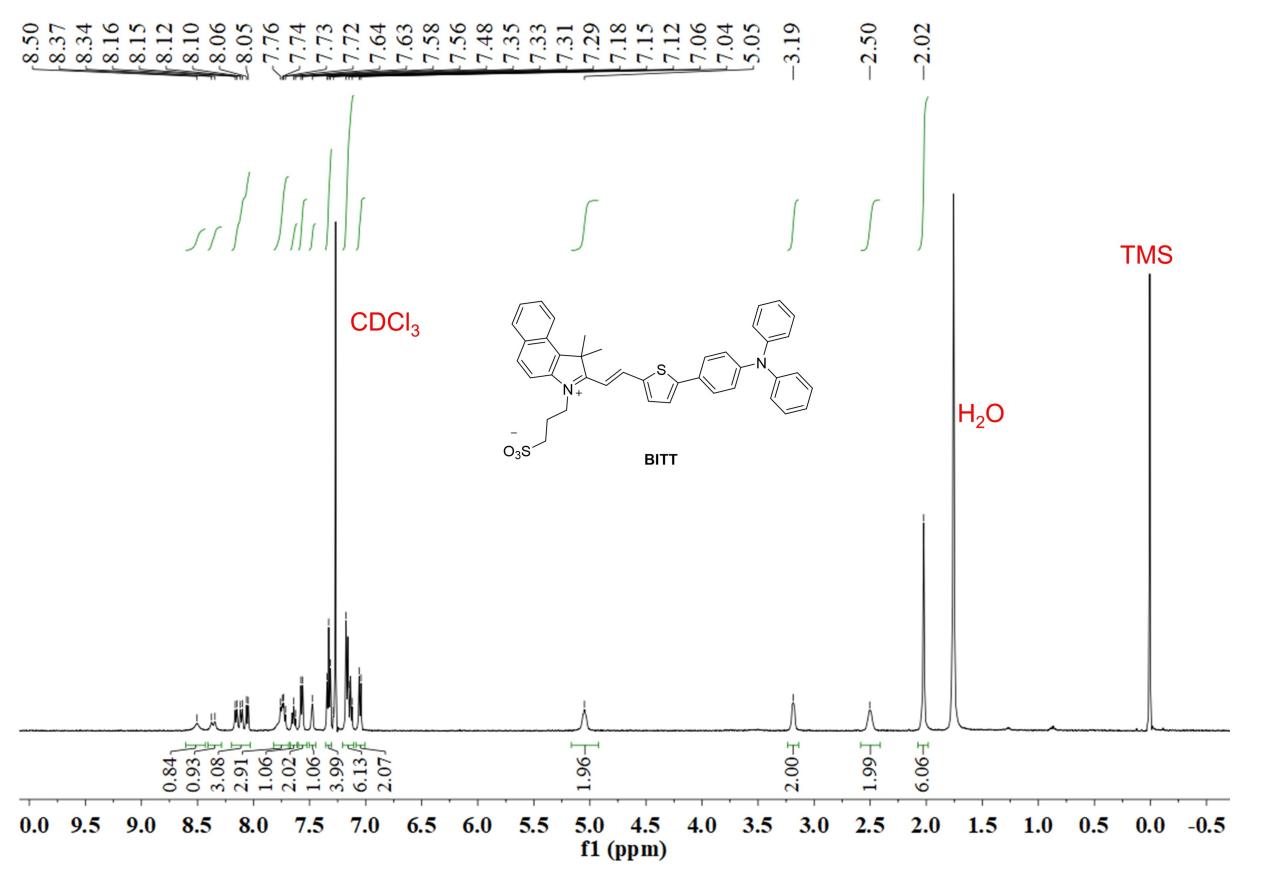
^

**Fig. S4** ^1^H NMR of BITT. ^1^H NMR (500 MHz, Chloroform-d) δ 8.50 (s, 1H), 8.36 (d, J=15.2 Hz, 1H), 8.20-8.02 (m, 3H), 7.83-7.70 (m, 3H), 7.63 (d, J=7.5 Hz, 1H), 7.57 (d, J=8.3 Hz, 2H), 7.48 (s, 1H), 7.33 (t, J=7.7 Hz, 4H), 7.21-7.11 (m, 6H), 7.05 (d, J=8.3 Hz, 2H), 5.05 (s, 2H), 3.19 (s, 2H), 2.50 (s, 2H), 2.02 (s, 6H).


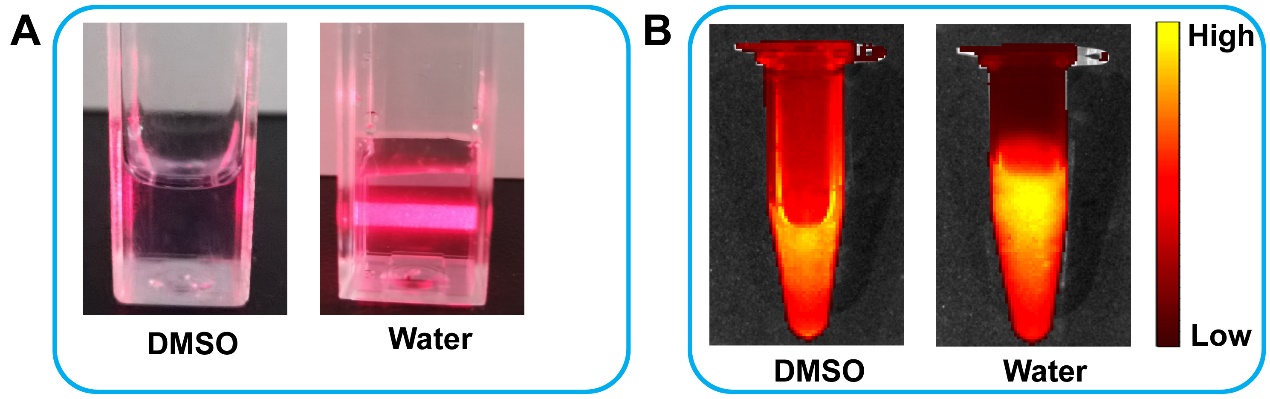


**Fig. S5.** The physical properties of BITT in different solutions. **A** The verification of Tyndall phenomenon. **B** Fluorescence imaging.


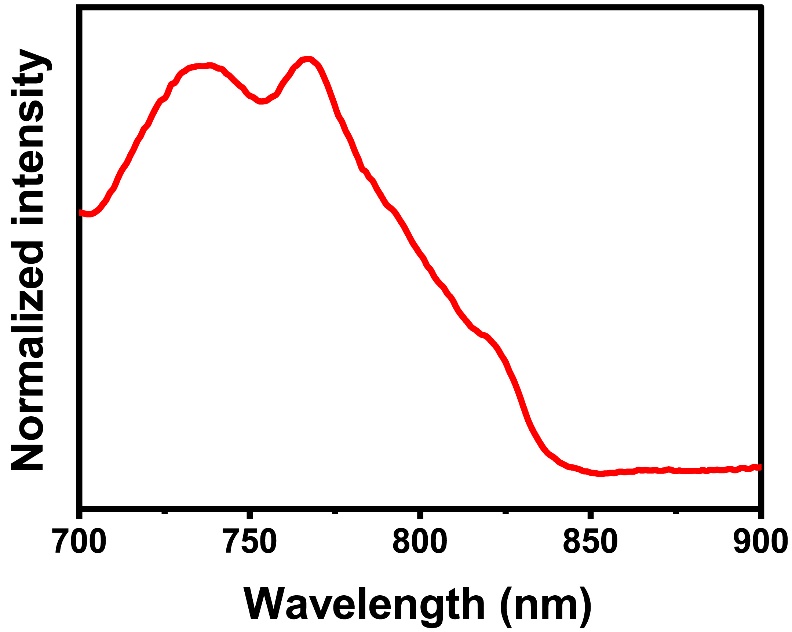


**Fig. S6** The emission spectrum in the presence of 660 nm irradiation.


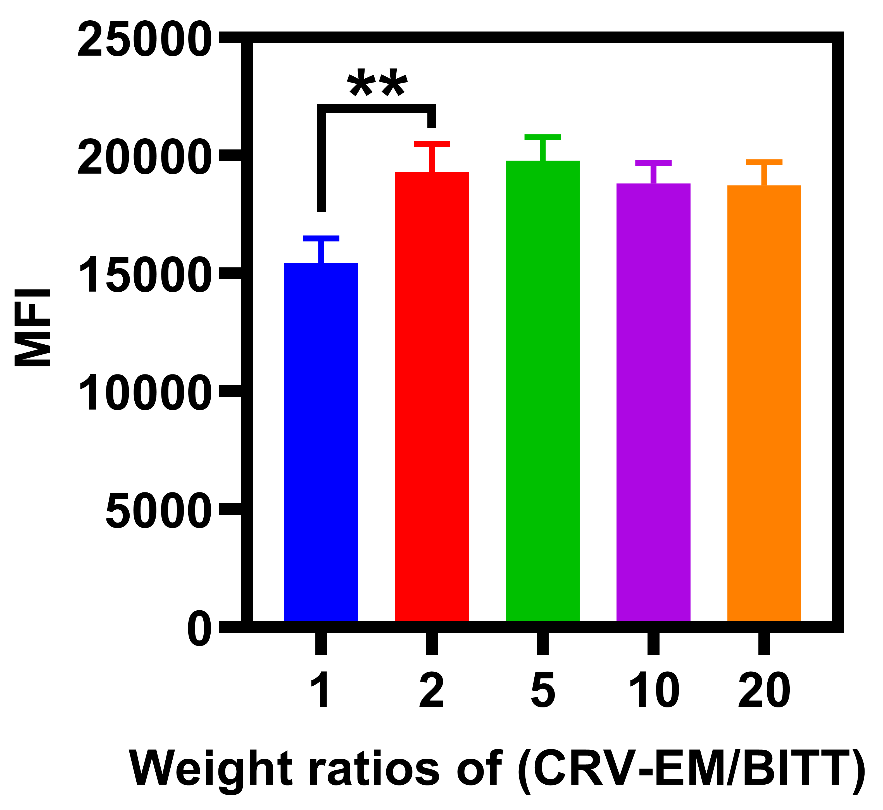


**Fig. S7** Mean fluorescence intensity (MFI) of the cells treated with CEB prepared with different weight ratios of CRV-EM to BITT.

**
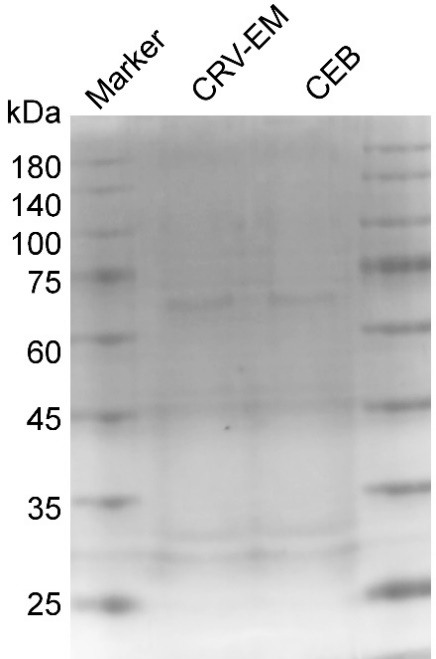
**

**Fig. S8** SDS-PAGE analysis of the retained proteins on the CEV-EM and CEB.


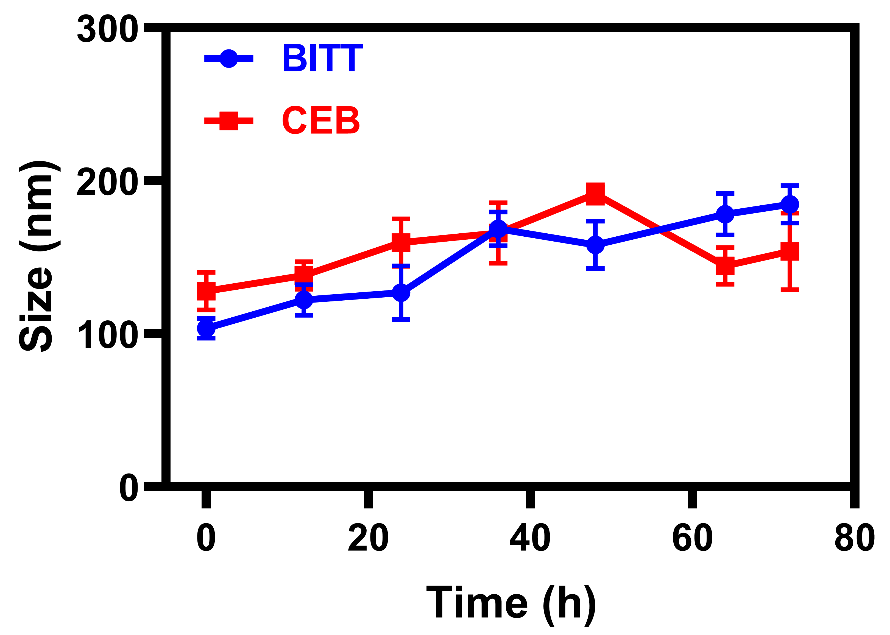


**Fig. S9** Stabilities of nanoparticles in PBS for 72 h.


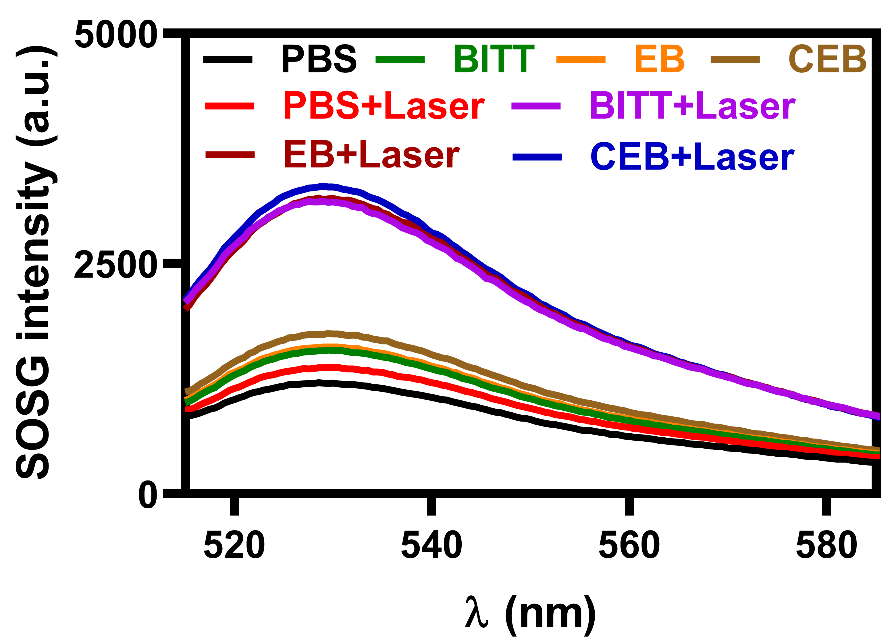


**Fig. S10** ROS level induced by the BITT-based nanoparticles.


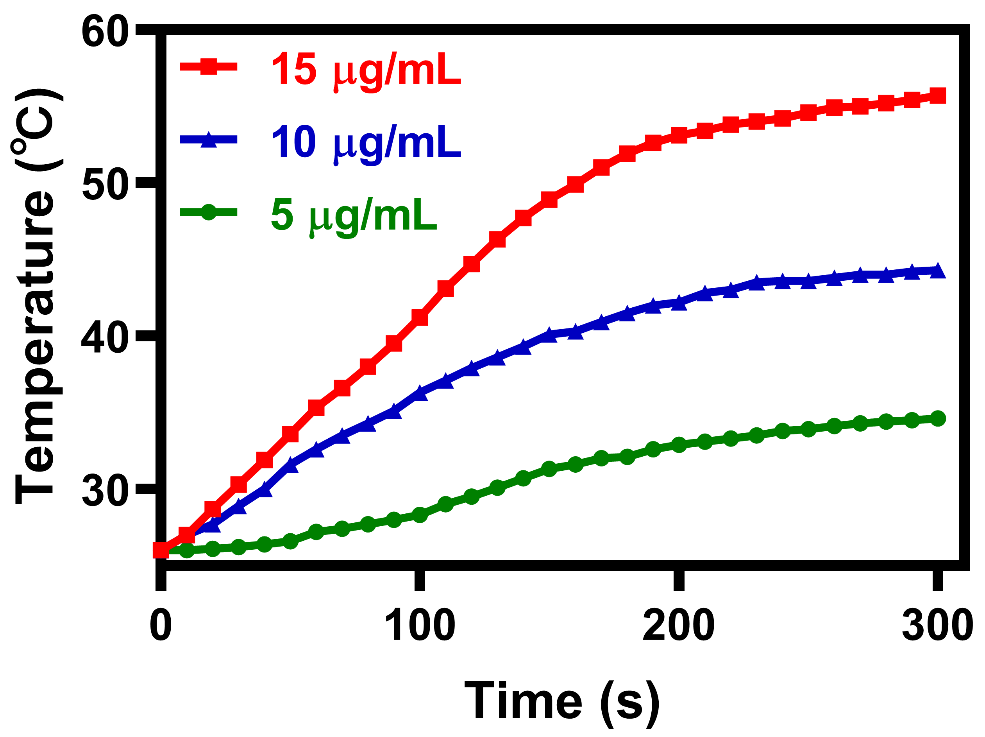


**Fig. S11** Photothermal conversion of CEB in different concentrations under 660 nm laser irradiation at the intensity of 2W/cm^2^.


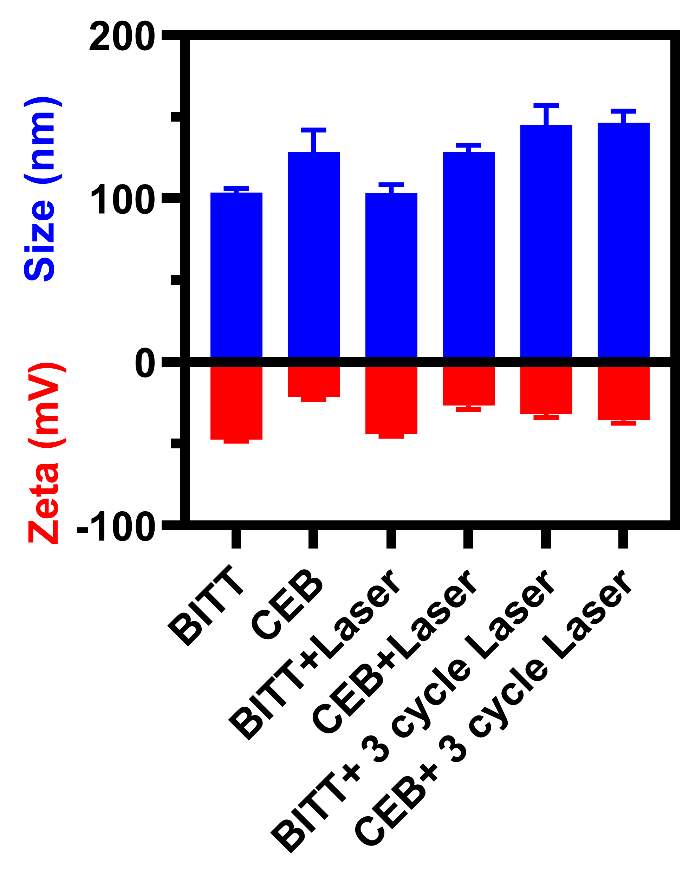


**Fig. S12** Size and zeta potential of the nanoparticles with or without laser irradiation.


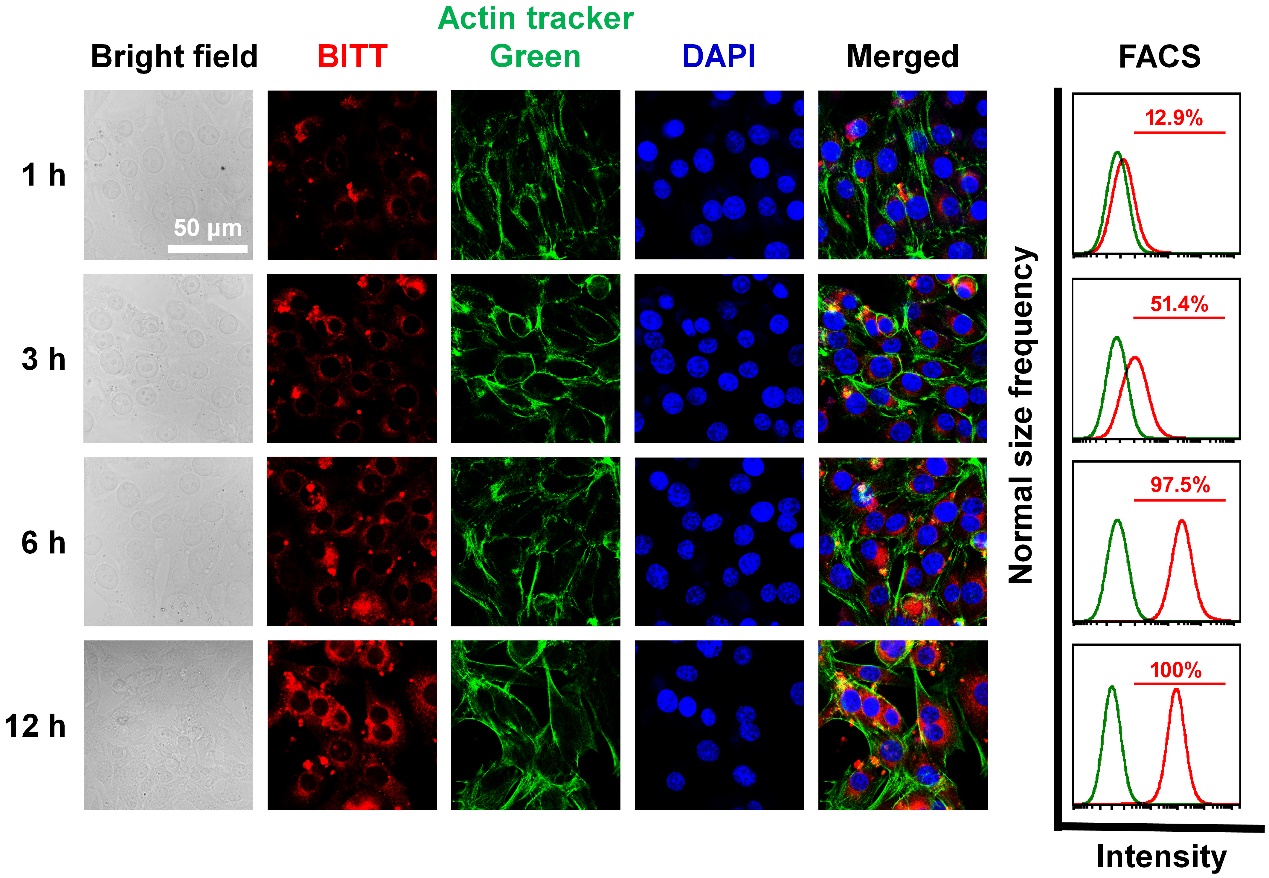


**Fig. S13** Cellular uptake of CEB in LLC cells incubated for different time.


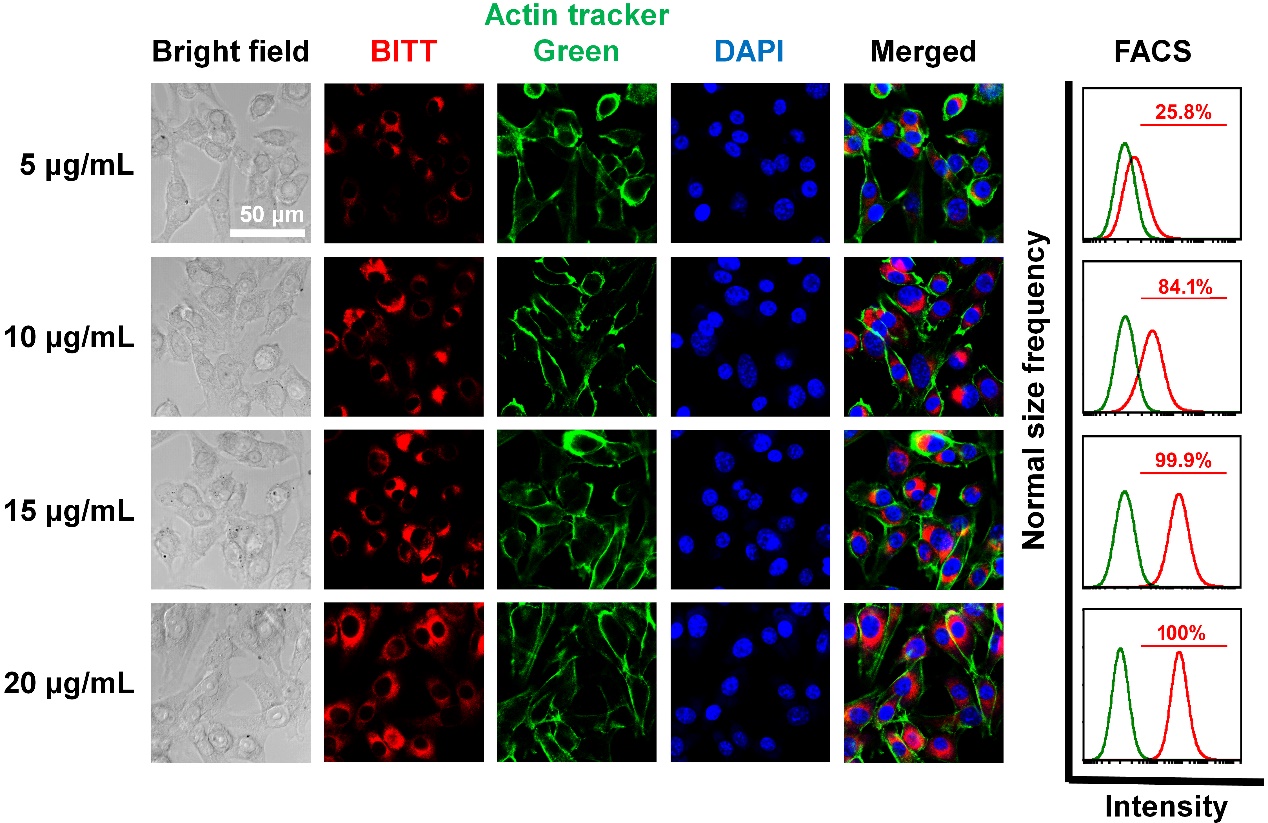


**Fig. S14** Cellular uptake of different concentrations CEB in LLC cells.


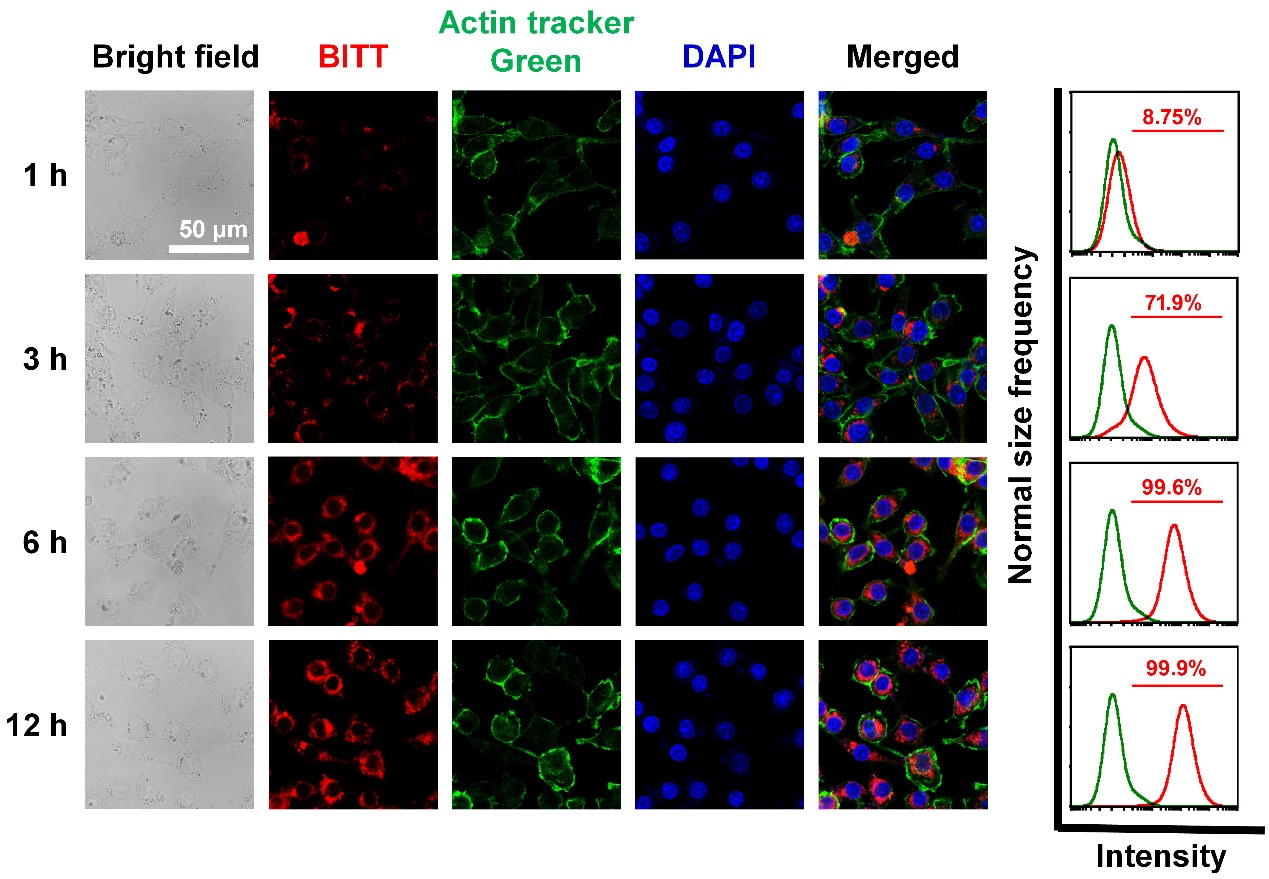


**Fig. S15** Cellular uptake of CEB in M2 macrophages incubated for different time.

**
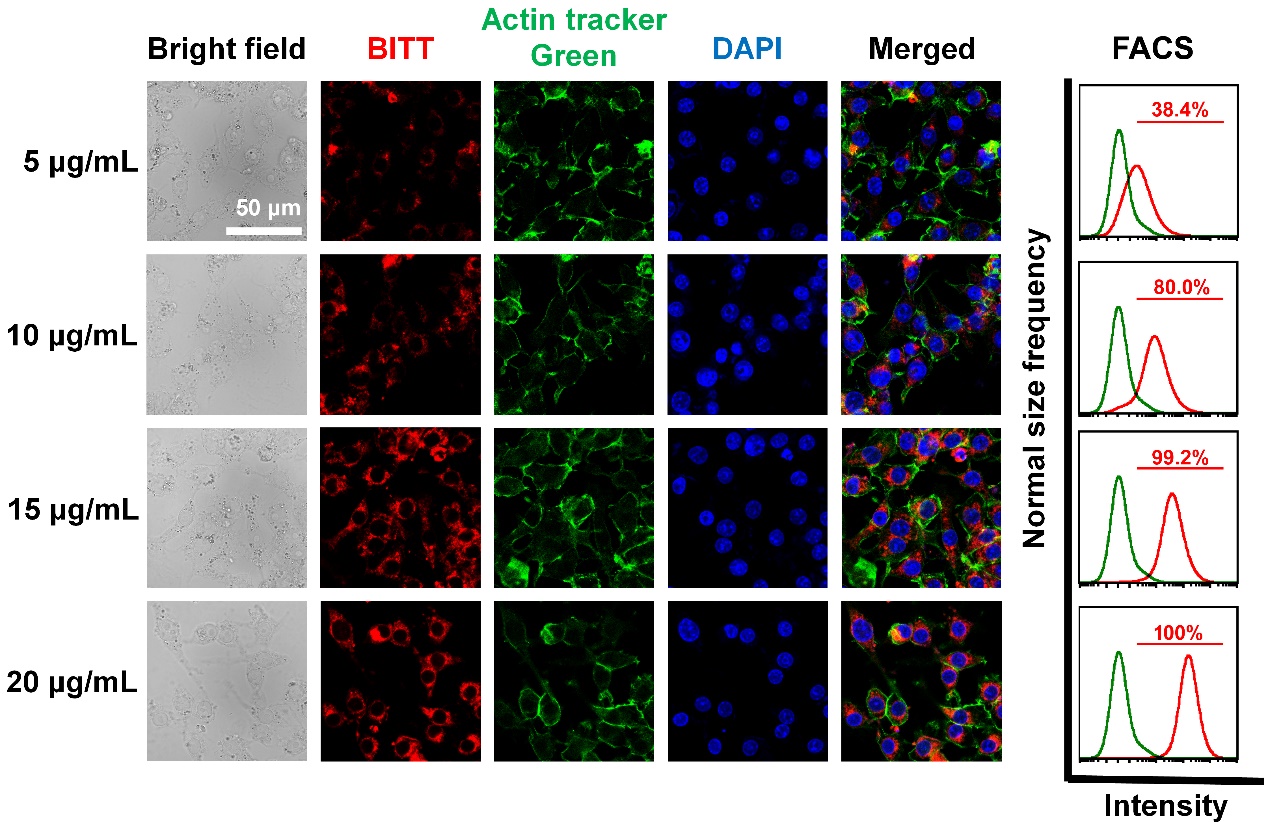
**

**Fig. S16** Cellular uptake of different concentrations CEB in M2 macrophages.

**
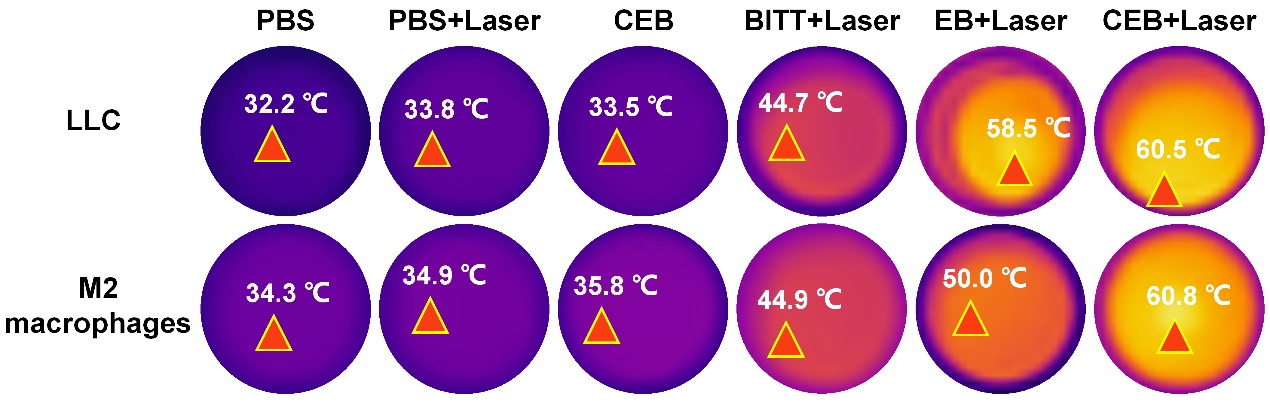
**

**Fig. S17** Photothermal effect induced by different formulations in cells.


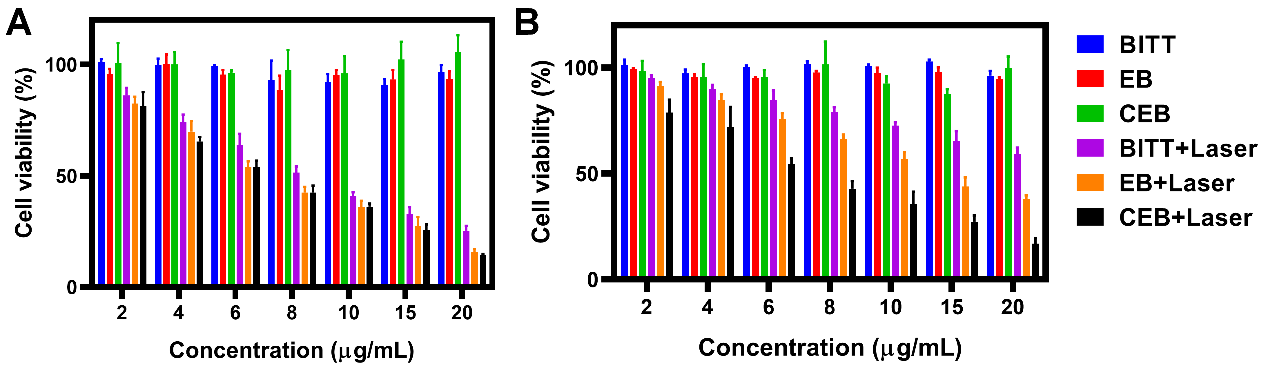


**Fig. S18** CCK8 analysis of cell viability. **A** LLC cells viability induced by BITT-based nanoparticles with/without irradiation. **B** M2 macrophages viability induced by BITT-based nanoparticles with/without irradiation.


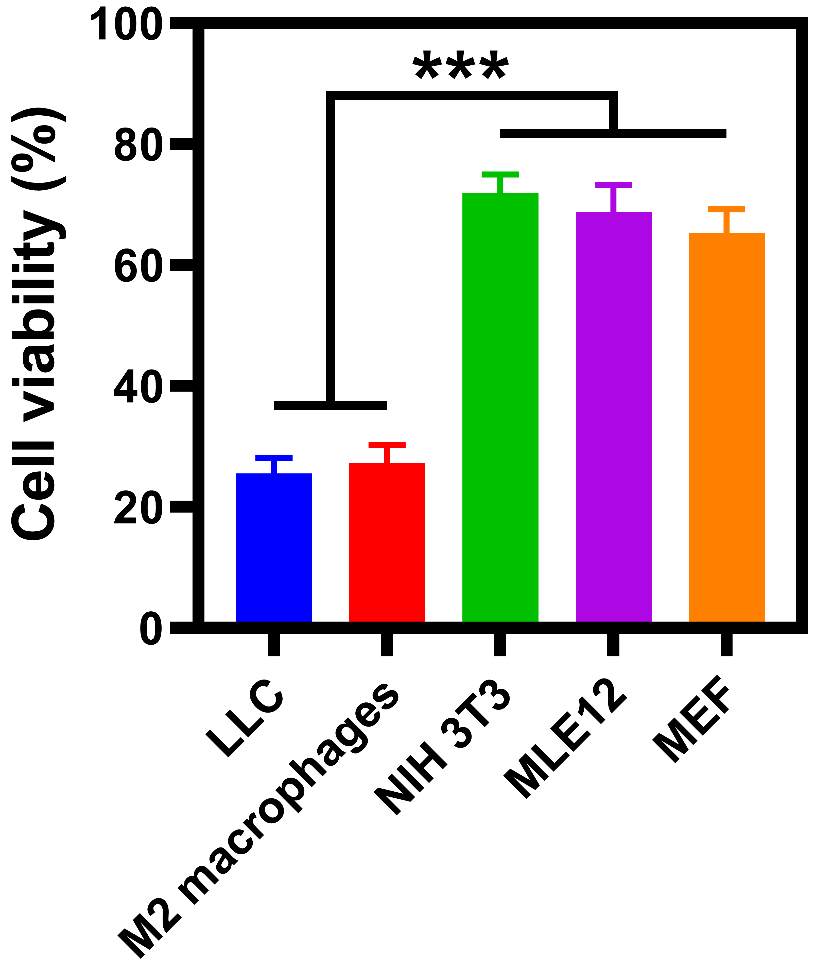


**Fig. S19** Cell viability of different cells induced by CEB. Different cell lines including LLC, M2 macrophages, NIH 3T3, MLE12, and MEF were treated with CEB at a BITT concentration of 15 μg/mL and exposed to the 660 nm irradiation for 5 min. CCK-8 assay was performed to test the cell viability.

**
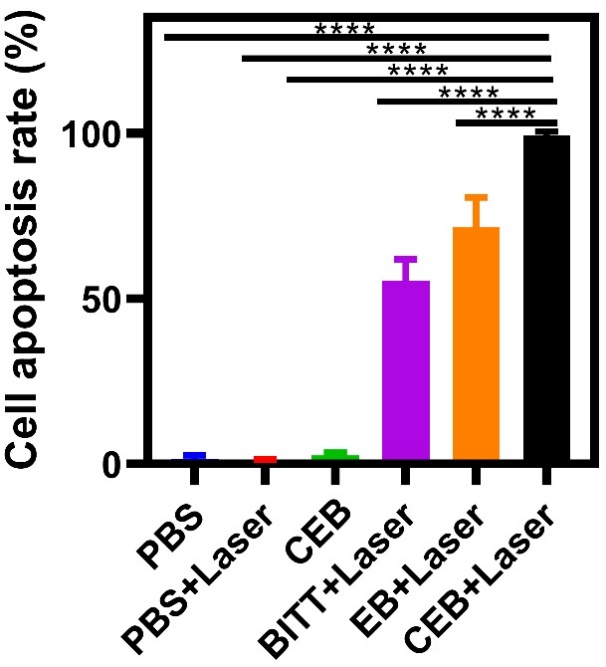
**

**Fig. S20** The quantitative analysis of the apoptosis in the tumors.

**
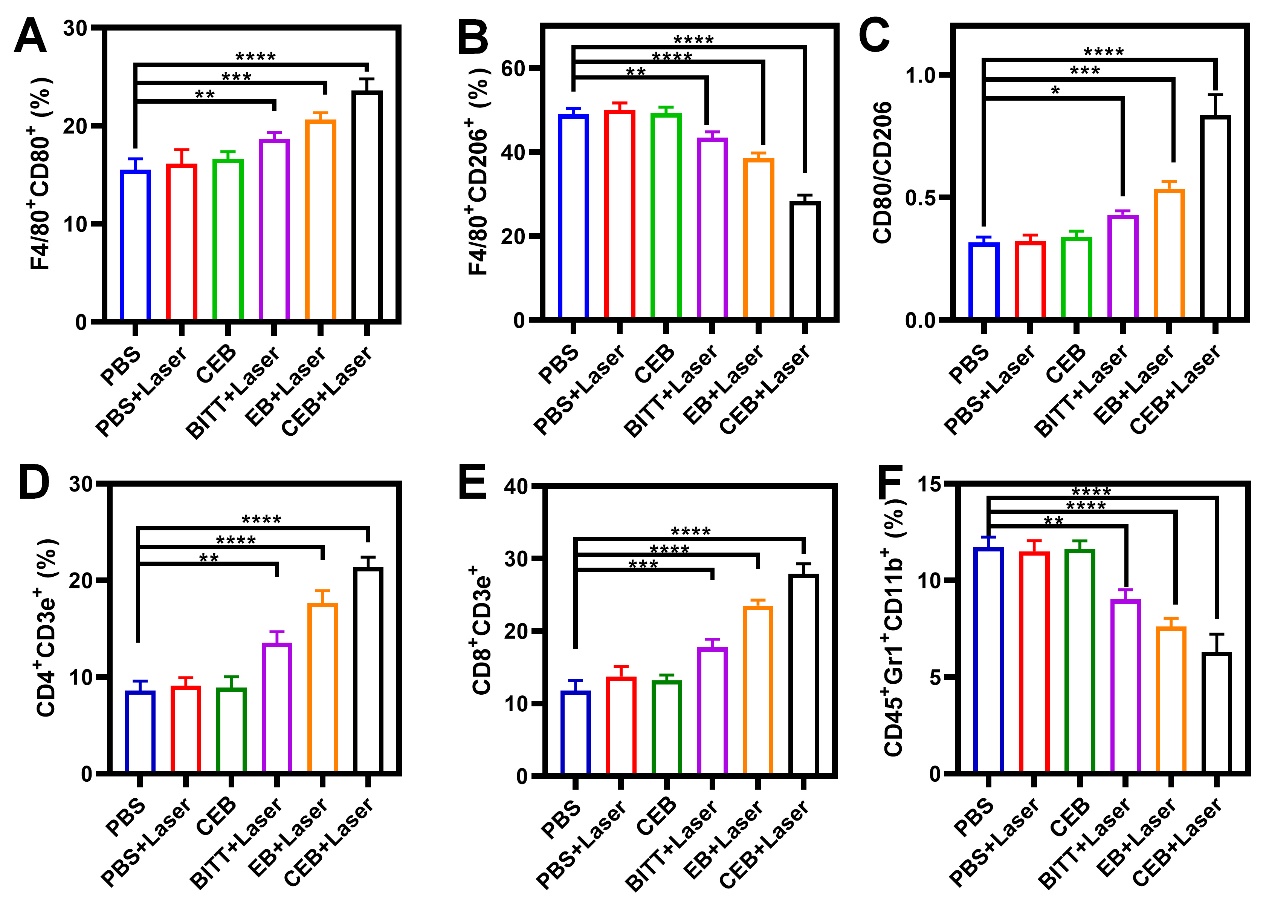
**

**Fig. S21** Quantitatively analysis of the immune cells after the treatment with different formulations. **A** M1 macrophages. **B** M2 macrophages. **C** The ratio of M1 to M2 macrophages. **D** CD8+ T cells. **E** CD4+ T cells. **F** MDSC cells.
